# Modulating the pharmacokinetic profile of Actinium-225-labeled macropa-derived radioconjugates by dual targeting of PSMA and albumin

**DOI:** 10.7150/thno.78043

**Published:** 2022-10-17

**Authors:** Falco Reissig, Kristof Zarschler, Zbynek Novy, Milos Petrik, Katerina Bendova, Daniela Kurfurstova, Jan Bouchal, Marie-Charlotte Ludik, Florian Brandt, Klaus Kopka, Marta Khoylou, Hans-Jürgen Pietzsch, Marian Hajduch, Constantin Mamat

**Affiliations:** 1 Helmholtz-Zentrum Dresden-Rossendorf, Institute of Radiopharmaceutical Cancer Research, Bautzner Landstraße 400, D‑01328 Dresden, Germany.; 2 Technische Universität Dresden, Faculty of Chemistry and Food Chemistry, D-01062 Dresden, Germany.; 3 Palacky University Olomouc, Faculty of Medicine and Dentistry, Institute of Molecular and Translational Medicine and Czech Advanced Technology and Research Institute, Hnevotinska 1333/5, 779 00 Olomouc, Czech Republic.; 4 Palacky University Olomouc, Faculty of Medicine and Dentistry, Institute of Clinical and Molecular Pathology, Hnevotinska 976/3, 775 15 Olomouc, Czech Republic.

**Keywords:** Macropa, Actinium, Targeted Alpha Therapy, Albumin Binder, PSMA

## Abstract

**Rationale:** Small ^225^Ac-labeled prostate-specific membrane antigen (PSMA)-targeted radioconjugates have been described for targeted alpha therapy of metastatic castration-resistant prostate cancer. Transient binding to serum albumin as a highly abundant, inherent transport protein represents a commonly applied strategy to modulate the tissue distribution profile of such low-molecular-weight radiotherapeutics and to enhance radioactivity uptake into tumor lesions with the ultimate objective of improved therapeutic outcome.

**Methods:** Two ligands **mcp-M-alb-PSMA** and **mcp-D-alb-PSMA** were synthesized by combining a macropa-derived chelator with either one or two lysine-ureido-glutamate-based PSMA- and 4-(*p*-iodophenyl)butyrate albumin-binding entities using multistep peptide-coupling chemistry. Both compounds were labeled with [^225^Ac]Ac^3+^ under mild conditions and their reversible binding to serum albumin was analyzed by an ultrafiltration assay as well as microscale thermophoresis measurements. Saturation binding studies and clonogenic survival assays using PSMA-expressing LNCaP cells were performed to evaluate PSMA-mediated cell binding and to assess the cytotoxic potency of the novel radioconjugates **[^225^Ac]Ac-mcp-M-alb-PSMA** and **[^225^Ac]Ac-mcp-D-alb-PSMA**, respectively. Biodistributions of both ^225^Ac-radioconjugates were investigated using LNCaP tumor-bearing SCID mice. Histological examinations of selected organs were performed to analyze the occurrence of necrosis using H&E staining, DNA damage via γH2AX staining and proliferation via Ki67 expression in the tissue samples.

**Results:** Enhanced binding to serum components in general and to human serum albumin in particular was revealed for **[^225^Ac]Ac-mcp-M-alb-PSMA** and **[^225^Ac]Ac-mcp-D-alb-PSMA**, respectively. Moreover, the novel derivatives are highly potent PSMA ligands as their K_D_ values in the nanomolar range (23.38 and 11.56 nM) are comparable to the reference radioconjugates **[^225^Ac]Ac-mcp-M-PSMA** (30.83 nM) and **[^225^Ac]Ac-mcp-D-PSMA** (10.20 nM) without albumin binders. The clonogenic activity of LNCaP cells after treatment with the ^225^Ac-labeled ligands was affected in a dose- and time-dependent manner, whereas the bivalent radioconjugate **[^225^Ac]Ac-mcp-D-alb-PSMA** has a stronger impact on the clonogenic cell survival than its monovalent counterpart **[^225^Ac]Ac-mcp-M-alb-PSMA**. Biodistribution studies performed in LNCaP tumor xenografts showed prolonged blood circulation times for both albumin-binding radioconjugates and a substantially increased tumor uptake (46.04 ± 7.77 %ID/g for **[^225^Ac]Ac-mcp-M-alb-PSMA** at 128 h p.i. and 153.48 ± 37.76 %ID/g at 168 h p.i. for **[^225^Ac]Ac-mcp-D-alb-PSMA**) with favorable tumor-to-background ratios. Consequently, a clear histological indication of DNA damage was discovered in the tumor tissues, whereas DNA double-strand break formation in kidney and liver sections was less pronounced.

**Conclusion:** The modification of the PSMA-based ^225^Ac-radioconjugates with one or two albumin-binding entities resulted in an improved radiopharmacological behavior including a greatly enhanced tumor accumulation combined with a rather low uptake in most non-targeted organs combined with a high excretion via the kidneys.

## Introduction

The specific delivery of alpha-particle emitting radionuclides to sites of locally advanced or metastatic disease represents the fundamental principle of targeted alpha therapy (TAT) in oncology 1. Due to their high-linear energy transfer (LET) and short path length of 50-100 μm 2, agents emitting alpha-particles are highly beneficial for the eradication of micrometastatic cancers and small-volume tumors as well as hematologic cancers and small clusters of cancer cells while sparing healthy cells 2. The particular strength of TAT is the spatially confined deposition of ionizing radiation leading to a high quantity of irreparable double strand breaks in the DNA of tumor cells and in doing so, to cell death 3-5.

Besides the already EMA and FDA-approved ^223^Ra applied as Xofigo® for palliative treatment of bone metastases in patients with prostate cancer 6, 7, there are few more alpha-emitting radionuclides to be potentially used in nuclear medicine 8-10. ^227^Th-labeled antibody conjugates are currently under evaluation 11, 12, as well as the idea of a ^212^Pb/^212^Bi *in vivo* generator is of emerging interest 13. Furthermore, ^149^Tb 14-16 and ^211^At 17-20 are also powerful candidates with appropriate radioconjugates being in clinical trials.

During the last decade, a lot of attention has been paid to the alpha emitter ^225^Ac due to its nearly ideal physical decay properties. The long half-life of 9.9 days and the decay chain with four alpha-particle emissions are the main reasons for the particular cytotoxicity of ^225^Ac and its impressive effectiveness in TAT 21. In contrast to heavy alkaline-earth metals, such as radium, the complexation of ^225^Ac and the formation of complexes with adequate stabilities 22 is feasible and can be achieved with the 12-membered macrocycle 1,4,7,10-tetraazacyclododecane-1,4,7,10-tetraacetic acid (DOTA) 23. However, DOTA chelation of large lanthanide ions such as actinium is kinetically slow and requires extensive heating as well as high ligand concentration for sufficient radiolabeling yields. The 18-membered macrocycle macropa and its NCS-functionalized pendant were described as exceptional chelators for ^225^Ac by Thiele *et al*. several years ago, allowing rapid complexation at room temperature 24. Based on these promising results, we recently developed and thoroughly evaluated two macropa-based PSMA (prostate-specific membrane antigen)-directed radioconjugates for TAT of metastatic castration-resistant prostate cancer (mCRPC) as part of a proof-of-concept study. These low-molecular-weight ligands showed substantial PSMA-specific accumulation in the tumor, rapid clearance from the organism through the kidneys and low nonspecific accumulation in non-targeted organs 25. The intentional introduction of albumin-binding units into low-molecular-weight radiopharmaceuticals represents a common strategy to enhance their blood circulation time and to increase their tumor uptake 26, 27. A longer blood retention time and extended bioavailability is highly beneficial for the treatment effectiveness especially for therapeutic radionuclides with longer half-lives such as ^225^Ac. During the last years, several albumin binders 28, *e.g.* functionalized lysines, ibuprofen 29 and 4-(*p*‑iodophenyl)butyric acid have been evaluated when combined with radiopharmaceuticals 30-34. In general, these studies indicate, that the introduction of albumin binders has positive effects on the biodistribution of radiotherapeutics and enhances their therapeutic efficacy 35.

Against this background, the present study aimed at improving the pharmacokinetics of our previously developed radioconjugates and at increasing their tumor uptake while maintaining efficient clearance from the blood to minimize radiation burden of healthy organs and tissues. Therefore, recently described alkyne-functionalized macropa chelators **mcp-M-click** and **mcp-D-click** 25 were functionalized resulting in conjugates with one PSMA- and one albumin-binding unit as well as a conjugate with two PSMA- and two albumin-binding units consisting of 4-(*p*-iodophenyl)butyrate. The conjugates were designed in accordance to the recently published ^177^Lu-HTK01169, which has been reported to possess a good biodistribution profile and therapeutic success in a xenograft model 36. Apart from radiolabeling and stability studies, these novel radioligands were evaluated *in vitro* regarding their interaction with serum components and human serum albumin. After investigating the binding behavior of the ^225^Ac-labeled compounds and their inhibitory effect on the clonogenicity of PSMA-expressing cells, tissue distribution studies were performed in LNCaP tumor-bearing SCID mice.

## Results and Discussion

### Conjugate Synthesis

**Scheme 1:**
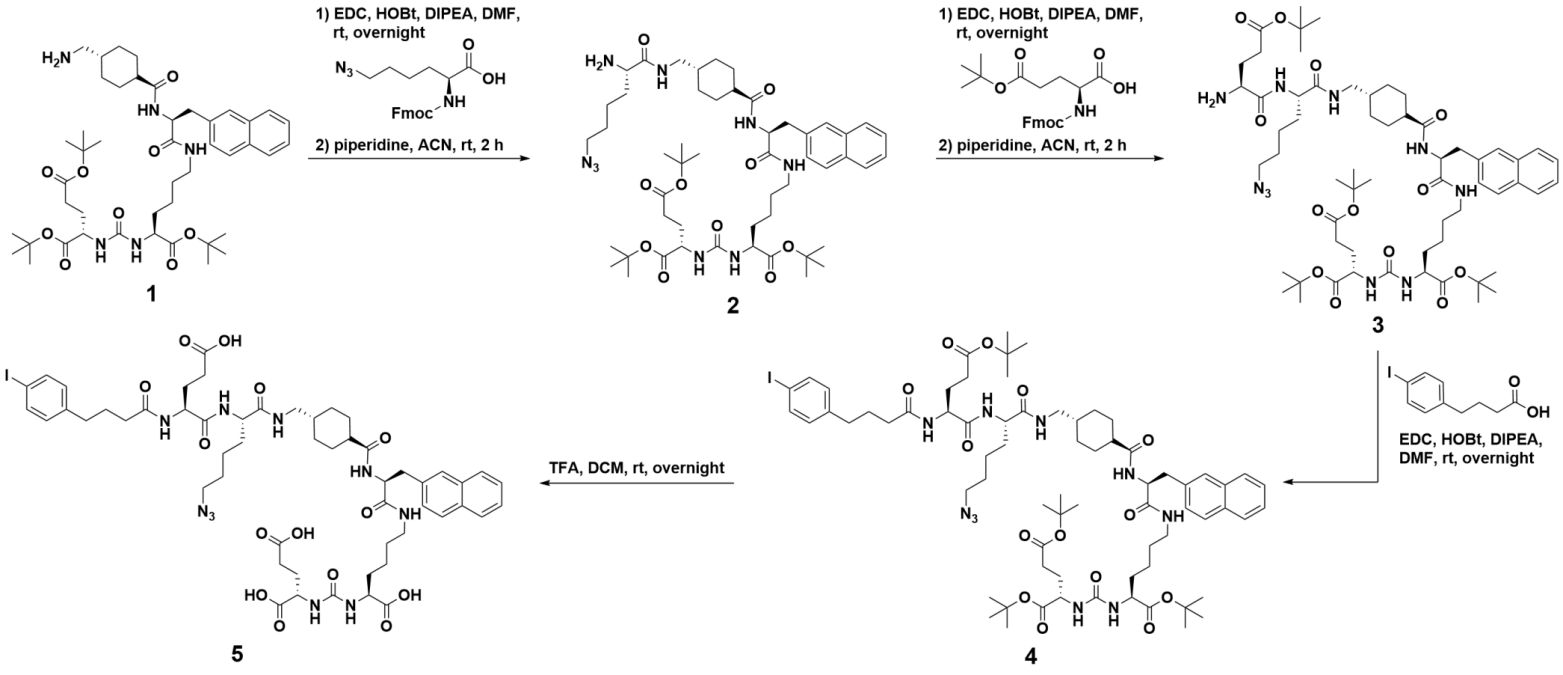
Synthesis path of the 4-(*p*-iodophenyl)butyrate-containing PSMA-derivative **5** starting from the ^t^Bu-protected PSMA-617-derived compound **1**.

**Scheme 2:**
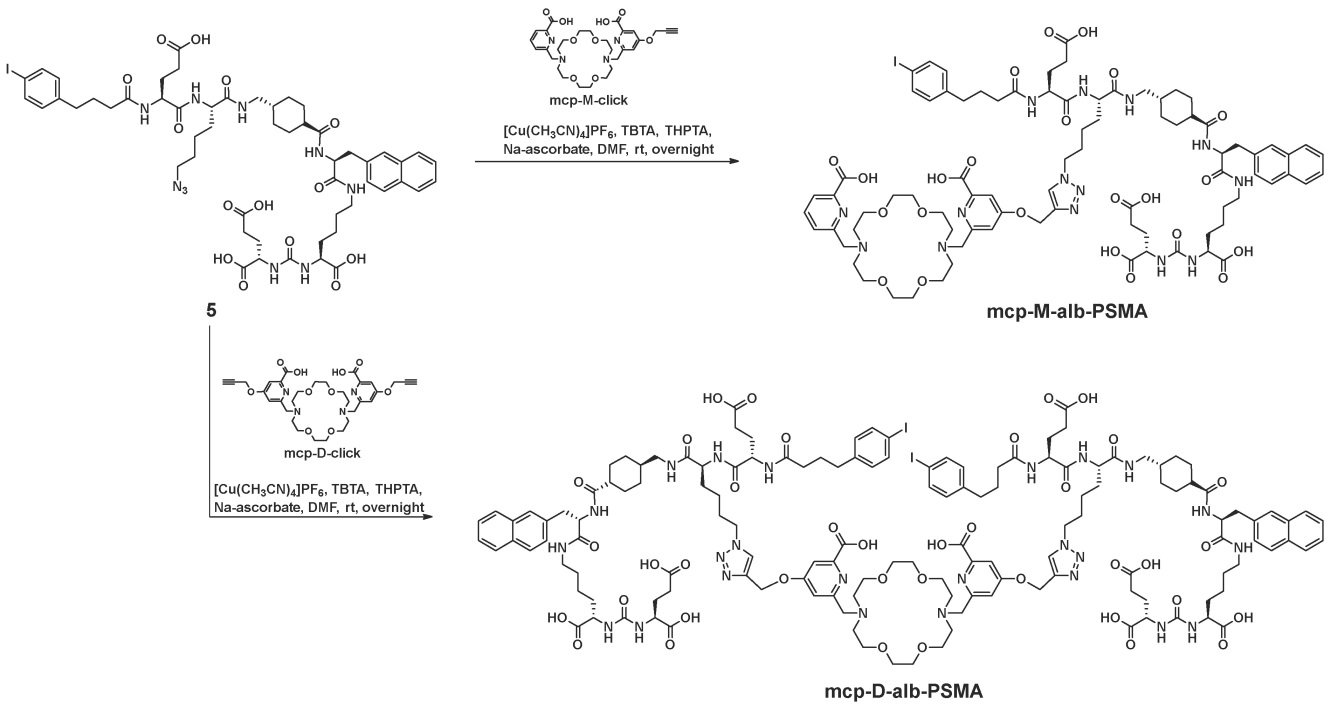
Synthesis of the both macropa-derivatives **mcp-M-alb-PSMA** and **mcp-D-alb-PSMA** from the alkyne-functionalized macropa chelators **mcp-M-click** and **mcp-D-cklick** using the CuAAC.

Following our recent study of therapeutic radiopharmaceuticals **[^225^Ac]Ac-mcp-M-PSMA** and **[^225^Ac]Ac-mcp-D-PSMA** with one and two PSMA binding moieties connected to a macropa-derived chelator to stably bind ^225^Ac 25, we have developed two new key derivatives with the same composition including additional 4-(*p*-iodophenyl)butyrate units as albumin binders. Whereas the synthesis of the both chelators **mcp-M-click** and **mcp-D-click** has been published elsewhere 25, the albumin-binding PSMA motif was built up by applying standard peptide coupling strategies followed by Fmoc-deprotection. Starting from the ^t^Bu-protected compound **1** with PSMA-binding motif present in PSMA-617 37, we synthesized the combined albumin and PSMA binding derivative **2** containing an additional branched azidolysine. The connection of a further glutamate unit led to compound **3**, which is basis for the introduction of the 4-(*p-*iodophenyl)butyrate as albumin binding entity leading to compound **4**. After deprotection, the desired compound **5** was obtained ready to be clicked (Scheme 1). The connection of the chelators **mcp-M-click** and **mcp-D-click** and the PSMA-binding biomolecule **5** has been realized via copper-mediated azide-alkyne-cycloaddition to yield the final conjugates **mcp-M-alb-PSMA** and **mcp-D-alb-PSMA**. The synthesis procedure is displayed in Scheme 2.

### Radiolabeling and Conjugate Stability

The final conjugates **mcp-M-alb-PSMA** and **mcp-D-alb-PSMA** were successfully radiolabeled with ^225^Ac at a concentration down to 10^-6^ M with radiochemical conversions of higher than 99% within 15 min at room temperature and a resulting molar activity of 1 MBq/nmol. The radiolabeled actinium complexes **[^225^Ac]Ac-mcp-M-alb-PSMA** and **[^225^Ac]Ac-mcp-D-alb-PSMA** were characterized regarding decomplexation (by radio-TLC) and degradation (by radio-HPLC). Both complexes did not show any ^225^Ac release over 7 days and no (72 h) or rather low (7 d) degradation, respectively (see Figures S8 and S9 for chromatograms). As recently shown 25, the addition of 2,5-dihydroxybenzoic acid to ^225^Ac-radioconjugates improved the long-term stability compared to a reference without additives. Log D values for **[^225^Ac]Ac-mcp-M-alb-PSMA** and **[^225^Ac]Ac-mcp-D-alb-PSMA** were determined at pH 7.4 to be ‑2.4 and -1.7, respectively.

Due to their excellent labeling properties, proven long-term stabilities, especially when 2,5-dihydroxybenzoic acid is applied as an additive and their sufficient hydrophilicity, both radiolabeled complexes **[^225^Ac]Ac-mcp-M-alb-PSMA** and **[^225^Ac]Ac-mcp-D-alb-PSMA** remain promising and underwent biological evaluation *in vitro* as well as *in vivo*.

### Protein Binding and Albumin Interaction

**Figure 1:**
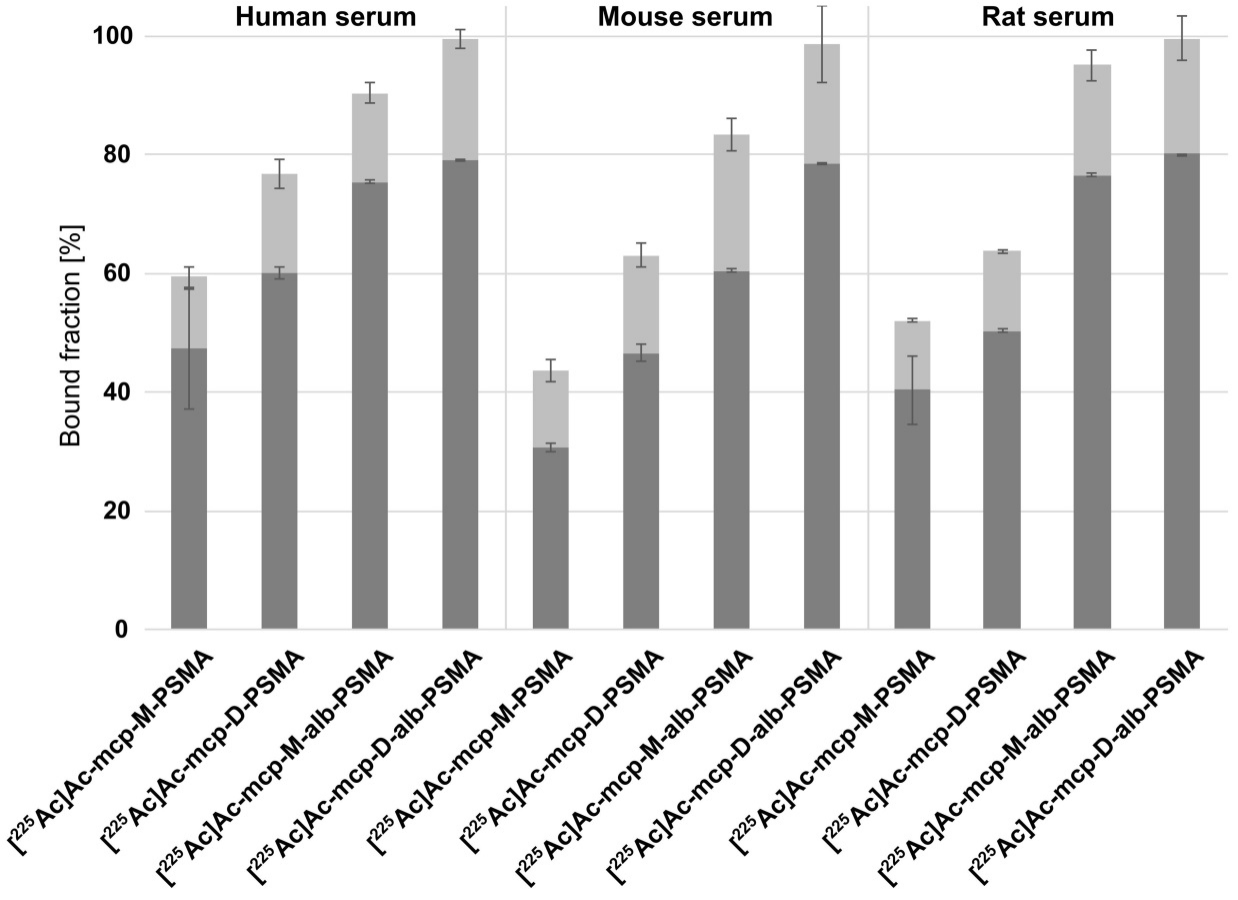
Serum binding properties of the 4-(p-iodophenyl)butyrate-containing radioconjugates **[^225^Ac]Ac-mcp-M-alb-PSMA** and **[^225^Ac]Ac-mcp-D-alb-PSMA** in comparison to **[^225^Ac]Ac-mcp-M-PSMA** and **[^225^Ac]Ac-mcp-D-PSMA** lacking an albumin-binding entity. The serum-bound fractions (dark gray) were calculated from the activity measured in the retained solutions relative to the total added activity. The fractions associated with the filter membranes after reverse spin are shown in light gray. The data are expressed as mean ± SD (n = 3).

The binding capacity of the new ^225^Ac-labeled radioconjugates **[^225^Ac]Ac-mcp-M-alb-PSMA** and **[^225^Ac]Ac-mcp-D-alb-PSMA** to mouse, rat and human serum proteins was determined by an ultrafiltration assay (Figure 1) in comparison with the previously developed radioconjugates **[^225^Ac]Ac-mcp-M-PSMA** and **[^225^Ac]Ac-mcp-D-PSMA** without albumin binder. All four radioconjugates show some degree of reversible binding to serum components and the percentage of retained activity is increasing with the molecular weight of the radioconjugates as well as with the number of 4-(*p-*iodophenyl)butyrate entities. The ^225^Ac-labeled PSMA-conjugates without albumin-binding moieties **[^225^Ac]Ac-mcp-M-PSMA** and **[^225^Ac]Ac-mcp-D-PSMA** show nonetheless a considerable binding to human and murine serum components (30-60%). However, a clearly increased binding to serum components in general was observed for the corresponding 4-(*p*-iodophenyl)butyrate-containing PSMA radioconjugates **[^225^Ac]Ac-mcp-M-alb-PSMA** and **[^225^Ac]Ac-mcp-D-alb-PSMA** with values between 60-80%. The observed differences between mouse, rat and human serum originate certainly from species-specific, structural deviations in the binding sites for 4-(*p*-iodophenyl)butyrate on albumin 38,39.

It is noteworthy that a certain fraction (12-23%) of each ^225^Ac-radioconjugate remains associated with the membrane of the ultrafiltration device despite reverse spinning presumably due to unspecific filter adsorption of the radioconjugates themselves or as a result of nonspecific filter interactions of protein-radioconjugate-complexes.

Ultrafiltration experiments performed with murine and human serum demonstrated the pronounced protein-binding properties of the 4-(*p*-iodophenyl)butyrate-containing PSMA radioconjugates. Additional microscale thermophoresis experiments with a solution of human serum albumin confirmed the transient interaction of these radioconjugates with this abundant transport protein (Table 1). Equilibrium dissociation constants (K_D_) of 27 µM and 15 µM were determined for **mcp-M-alb-PSMA** and **mcp-D-alb-PSMA** in competition for the 4-(*p-*iodophenyl)butyrate-binding site (Sudlow Site II). The lower K_D_ value of the bivalent compound **mcp-D-alb-PSMA** originates from the presence two independent 4-(*p-*iodophenyl)butyrate moieties resulting in a higher avidity compared to **mcp-M-alb-PSMA**. In contrast to the 4-(*p*-iodophenyl)butyrate-modified conjugates, the analogs **mcp-M-PSMA** and **mcp-D-PSMA** show a much lower albumin-binding affinity with K_D_ values of 330 µM and 150 µM, respectively. This data is in accordance with the results of the ultrafiltration assay, where the PSMA radioconjugates without dedicated albumin binder show certain binding to serum proteins as well. This behavior has been likewise described for a range of PSMA ligands 29,31 and can be attributed to the interaction of their naphthyl entities with the 2-naphthylamine binding sites in serum albumin 40.

### Characterization of the PSMA-binding properties

**Figure 2:**
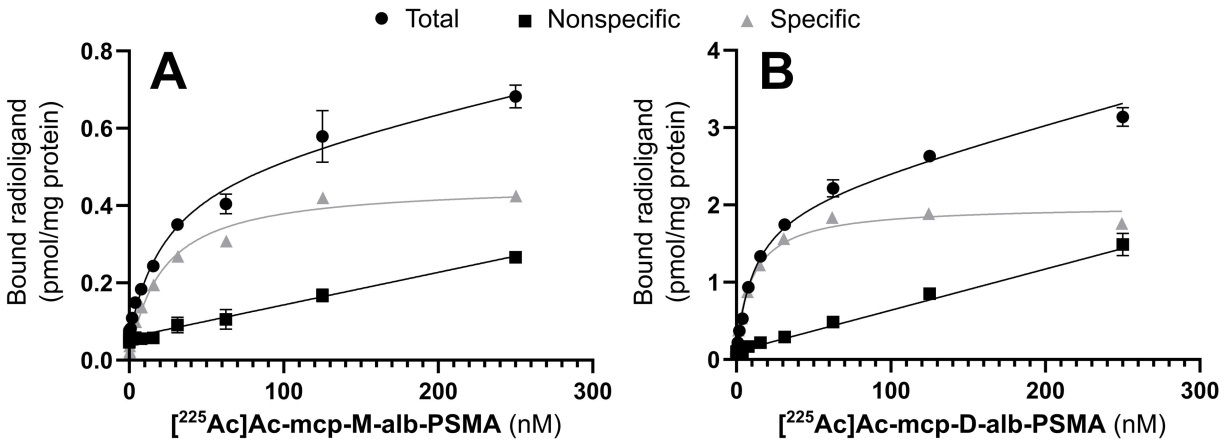
Saturation binding curves for (A) **[^225^Ac]Ac-mcp-M-alb-PSMA** and (B) **[^225^Ac]Ac-mcp-D-alb-PSMA**. Nonspecific binding was determined in the presence of 500 µM unlabeled PSMA-617. Specific binding was calculated as the difference between total and nonspecific binding (n = 3 for each data point).

The cell binding behavior of the ^225^Ac-labeled, 4-(*p*-iodophenyl)butyrate-containing PSMA radioconjugates **[^225^Ac]Ac-mcp-M-alb-PSMA** and **[^225^Ac]Ac-mcp-D-alb-PSMA** was analyzed in saturation binding studies using PSMA-positive LNCaP cells. The corresponding equilibrium dissociation constants K_D_ and maximum binding capacities B_max_ are summarized in Table 1, and representative saturation binding curves are given in Figure 2.

The introduction of the albumin-binding entities has no substantial influence on PSMA binding as the K_D_ values of the 4-(*p*-iodophenyl)butyrate-functionalized radioconjugates are comparable to the reference radioconjugates **[^225^Ac]Ac-mcp-M-PSMA** and **[^225^Ac]Ac-mcp-D-PSMA** without dedicated albumin binder.

Interestingly, the maximum binding capacities (B_max_) of the bivalent radioconjugates (**[^225^Ac]Ac-mcp-D-PSMA** and **[^225^Ac]Ac-mcp-D-alb-PSMA**) are higher than the values of the corresponding monovalent derivatives (**[^225^Ac]Ac-mcp-M-PSMA** and **[^225^Ac]Ac-mcp-M-alb-PSMA**) indicating, that a higher amount of the bivalent ligands is binding per mg of protein, although the absolute number of potential binding sites at the cell surface is constant. One reason for this difference in the B_max_ values might be the dissimilarity of the radioligand's polarity 41 as the hydrophilic monovalent derivatives have a lower maximum binding capacity then the more lipophilic bivalent ones.

### Clonogenicity in response to incubation with ^225^Ac-labeled PSMA derivatives

**Figure 3:**
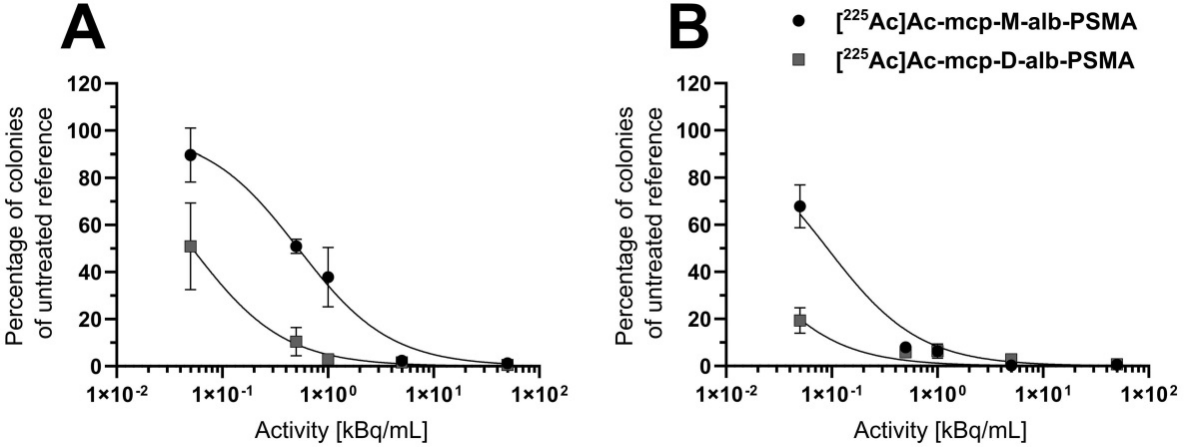
Clonogenic survival of LNCaP cells after treatment with **[^225^Ac]Ac-mcp-M-alb-PSMA** or **[^225^Ac]Ac-mcp-D-alb-PSMA** for (A) 1 h or (B) 4 h, respectively. Cells were exposed to different activity concentrations of 225Ac-labeled PSMA conjugates for the indicated treatment time and thereafter supplemented with fresh medium. Eight days later, colonies formed were stained and counted by digital analysis. Data points represent the mean colony number of three samples, normalized to values obtained for the untreated reference samples.

The cytotoxic activities of both ^225^Ac-labeled, 4-(*p*-iodophenyl)butyrate-containing PSMA radioconjugates were examined in clonogenic survival assays with LNCaP cells (Figure 3 and Table 1), and representative images of colonies exposed to increasing activity concentrations are shown in Figure S10 of the SI.

A time- and dose-dependent decrease in the clonogenic activity of the human prostate adenocarcinoma cells was observed for both radioconjugates (Figure 3). The derivative **[^225^Ac]Ac-mcp-M-alb-PSMA** with one PSMA-binding motif blocked colony outgrowth by about 50% at an activity concentration of 0.5 kBq/mL after 1 h exposure time, with an almost complete inhibition of colony formation observed at a concentration of 5 kBq/mL. At the same exposure time, its counterpart **[^225^Ac]Ac-mcp-D-alb-PSMA** with two PSMA-binding motifs shows an approximately 50% inhibition on colony formation already at the lowest dose of 0.05 kBq/mL and virtually abrogated clonogenic activity at a concentration of 0.5 kBq/mL. A longer treatment time resulted in an even more severe suppression of clonogenic survival mediated by the ^225^Ac-labeled conjugates. In agreement with corresponding data obtained for the PSMA radioconjugates without dedicated albumin binder (**[^225^Ac]Ac-mcp-M-PSMA** and **[^225^Ac]Ac-mcp-D-PSMA**) 25, the bivalent **[^225^Ac]Ac-mcp-D-alb-PSMA** outperforms its monovalent counterpart **[^225^Ac]Ac-mcp-M-alb-PSMA** in terms of cell killing capacity and antiproliferative activity.

### Small Animal Biodistribution

**Figure 4:**
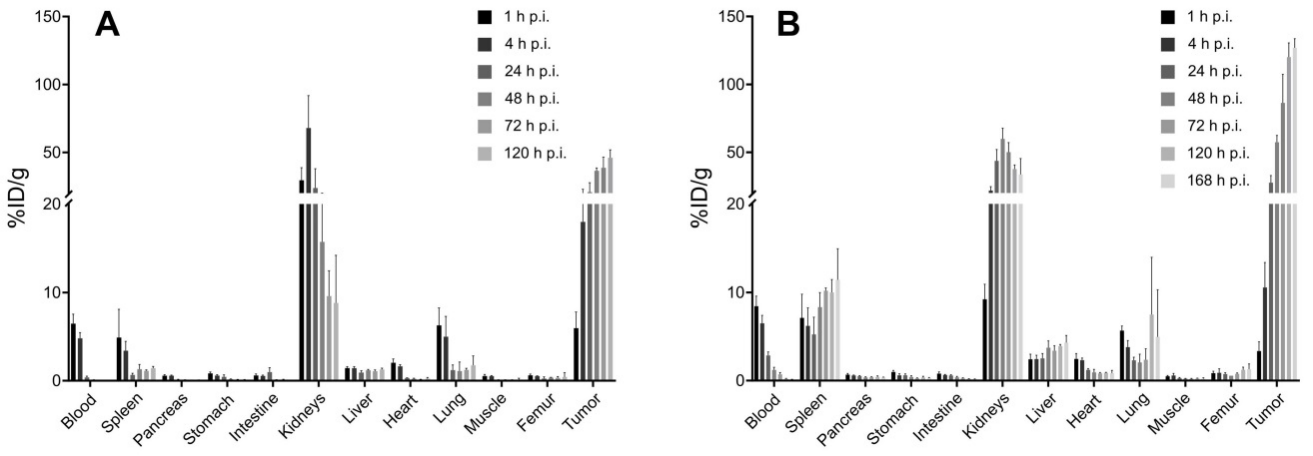
Biodistribution of (A) **[^225^Ac]Ac-mcp-M-alb-PSMA** and (B) **[^225^Ac]Ac-mcp-D-alb-PSMA** using LNCaP tumor-bearing SCID mice from 1 h to 120 resp. 168 h p.i. The data are presented as the mean of the percentage of injected dose per gram of organ ± standard deviation (n = 4 for the graph A and n = 3 for the graph B).

The biodistribution data of **[^225^Ac]Ac-mcp-M-alb-PSMA** and **[^225^Ac]Ac-mcp-D-alb-PSMA** showed similar *in vivo* behavior for both radioconjugates in LNCaP tumor-bearing mice. Complete *ex vivo* data are presented in graphs in Figure 4. The highest accumulation of radioactivity was found in the LNCaP tumors at 168 h p.i. for **[^225^Ac]Ac-mcp-D-alb-PSMA** (153.48 ± 37.76 %ID/g) and at 128 h p.i. for **[^225^Ac]Ac-mcp-M-alb-PSMA** (46.04 ± 7.77 %ID/g), whereas the gold standard in this field [^177^Lu]Lu-PSMA-617 possess tumor uptake of 11.20 ± 4.17 %ID/g (24 h p.i.) 42. Higher tumor uptake of **[^225^Ac]Ac-mcp-D-alb-PSMA** compared to **[^225^Ac]Ac-mcp-M-alb-PSMA** is in accordance with its higher PSMA-binding properties which are presented in Table 1. When comparing the maximal tumor uptake of **[^225^Ac]Ac-mcp-M-alb-PSMA** and **[^225^Ac]Ac-mcp-D-alb-PSMA** with their counterparts without dedicated albumin binders 25, an approximately four times higher tumor uptake was found for **[^225^Ac]Ac-mcp-M-alb-PSMA** compared to **[^225^Ac]Ac-mcp-M-PSMA** and an even twelve times higher tumor uptake for **[^225^Ac]Ac-mcp-D-alb-PSMA** compared to **[^225^Ac]Ac-mcp-D-PSMA**.

The second most prominent organ of radioconjugate uptake are kidneys with the highest accumulation of 67.92 ± 20.67 %ID/g (4 h p.i.) for **[^225^Ac]Ac-mcp-M-alb-PSMA** and 59.90 ± 6.46 %ID/g (48 h p.i.) for **[^225^Ac]Ac-mcp-D-alb-PSMA**. The presence of albumin binder in the structure of the radioconjugate caused a significantly longer kidney retention compared to the previously published data for the analogous radioconjugates without albumin binders 25 and for [^177^Lu]Lu-PSMA-617 42. Especially in the case of **[^225^Ac]Ac-mcp-D-alb-PSMA,** a high accumulation in the kidneys was found with 33.67 ± 9.57 %ID/g even at 168 h p.i. In contrast, there was only 8.82 ± 4.40 %ID/g of **[^225^Ac]Ac-mcp-M-alb-PSMA** retaining in kidneys at 120 h p.i. This feature makes **[^225^Ac]Ac-mcp-M-alb-PSMA** probably a more suitable candidate for further development than its counterpart with two PSMA and albumin binding motifs. The blood clearance of both radioconjugates with 4-(*p*-iodophenyl)butyrate moieties was slower compared to **[^225^Ac]Ac-mcp-M-PSMA** and **[^225^Ac]Ac-mcp-D-PSMA**, where the majority of the activity was cleared within first 4 to 24 h after the administration, meanwhile in case of **[^225^Ac]Ac-mcp-M-alb-PSMA** and **[^225^Ac]Ac-mcp-D-alb-PSMA** the excretion lasted 24 h and 72 h, respectively. The other examined organs revealed negligible radioactivity uptake except the spleen in the case of **[^225^Ac]Ac-mcp-D-alb-PSMA** with a maximal uptake of 11.42 ± 2.89 %ID/g. The potential long-term radiotoxic effects in non-targeted organs especially the kidneys and spleen should be the subject of further studies with both albumin-binding PSMA radioligands.

Tumor-to-background ratios were favorably high for both tested compounds and increased rapidly with time after the administration, especially from 24 h p.i. and further. Tumor-to-blood ratios at longest time intervals were calculated at 1490 and 1419 for **[^225^Ac]Ac-mcp-M-alb-PSMA** and **[^225^Ac]Ac-mcp-D-alb-PSMA**, respectively.

Non-decay-corrected data from the *ex vivo* biodistribution study were used to calculate the areas under the curves (AUCs) for the uptake of the radioligands in the blood pool, tumors, kidneys and the liver (Table 2). **[^225^Ac]Ac-mcp-D-alb-PSMA** showed almost double AUC for tumor accumulation compared to its monovalent counterpart. Similar situation is for blood, liver and kidneys AUCs values, what results in very similar AUCs ratios for both tested radioligands with the exception of AUC_Tu_-to-AUC_Li_, where **[^225^Ac]Ac-mcp-D-alb-PSMA** has 0.7-fold lower ratio compared with monovalent version of the ligand. The tumor AUCs for **[^225^Ac]Ac-mcp-M-alb-PSMA** and** [^225^Ac]Ac-mcp-D-alb-PSMA** are comparable with values for [^177^Lu]Lu-PSMA-617 and [^177^Lu]Lu-PSMA-alb variants, respectively, published by Benesova et al. 31.

### Histological examination of tumor, kidney and liver

**Figure 5:**
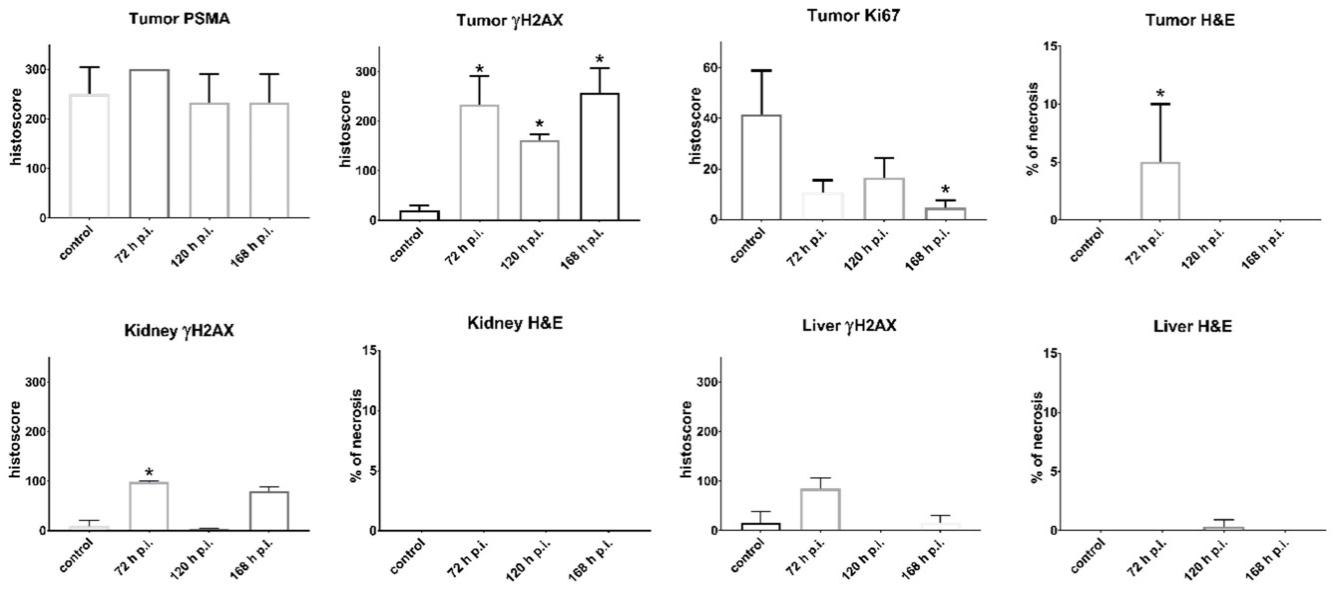
Histological assessment of organ sections. The graphs in the first row describe quantification of the corresponding Immunohistochemical (IHC) staining of the tumor tissue from the control group (untreated) and from groups injected with **[^225^Ac]Ac-mcp-D-alb-PSMA**. IHC staining confirmed high PSMA expression, γH2AX showed high DNA damage in the tumor tissue, while Ki67 displayed decrease in the tumor cell proliferation and H&E staining revealed necrotic lesions in the group 72 h p.i. Values are expressed as mean with standard deviations. P-values p < 0.05 are indicated with *. n = 6 for control groups and n= 3 for **[^225^Ac]Ac-mcp-D-alb-PSMA** treated groups.

The results of histological examination are summarized in Figure 5 for mice injected with **[^225^Ac]Ac-mcp-D-alb-PSMA**. As the results from **[^225^Ac]Ac-mcp-M-alb-PSMA** mice are very similar, they are attached in the Supplementary Information (see Figure S15). Immunohistochemical (IHC) staining of tumor tissue confirmed very high PSMA expression of the tumor cells what is typical for LNCaP tumor model 43. The γH2AX staining revealed significant DNA damage in tumor cells of mice treated with **[^225^Ac]Ac-mcp-M-alb-PSMA** as well as **[^225^Ac]Ac-mcp-D-alb-PSMA** compared to untreated controls, indicating the effect of both ^225^Ac-radioconjugates on double strand breaks in DNA. Nevertheless, the cellular proliferation investigated via Ki67 expression did not show any significant decrease in proliferation of the tumor cells except the 168 h p.i. group injected with **[^225^Ac]Ac-mcp-D-alb-PSMA**. Such low impact of ^225^Ac-radioconjugates on the proliferation of the cells is probably connected with typical low proliferation of prostate tumors 44. Hematoxylin and eosin stain (H&E) of the tumor tissue displayed slight but still significant necrosis only for the samples from 72 h p.i. time interval, meanwhile other investigated time intervals (120 h and 168 h p.i.) were practically necrosis free. This may suggest that recorded necrotic spots at 72 h p.i. does not have to be necessarily connected with the application of the tested ^225^Ac-radioconjugates.

The γH2AX staining of the kidney tissue showed insignificant DNA damage in this organ compared to the untreated controls. The only exception were kidney samples from 72 h p.i. time intervals of both evaluated ^225^Ac-conjugates, where average γH2AX histoscore were 62 for **[^225^Ac]Ac-mcp-M-alb-PSMA** and 99 for **[^225^Ac]Ac-mcp-D-alb-PSMA**, respectively. However, the DNA damage was much higher in the tumor tissue of these mice, resulting in γH2AX histoscores ranging from 149 up to 257, which were more than double of the renal ones. H&E stain did not show any necrosis in the examined kidney tissues.

**Figure 6:**
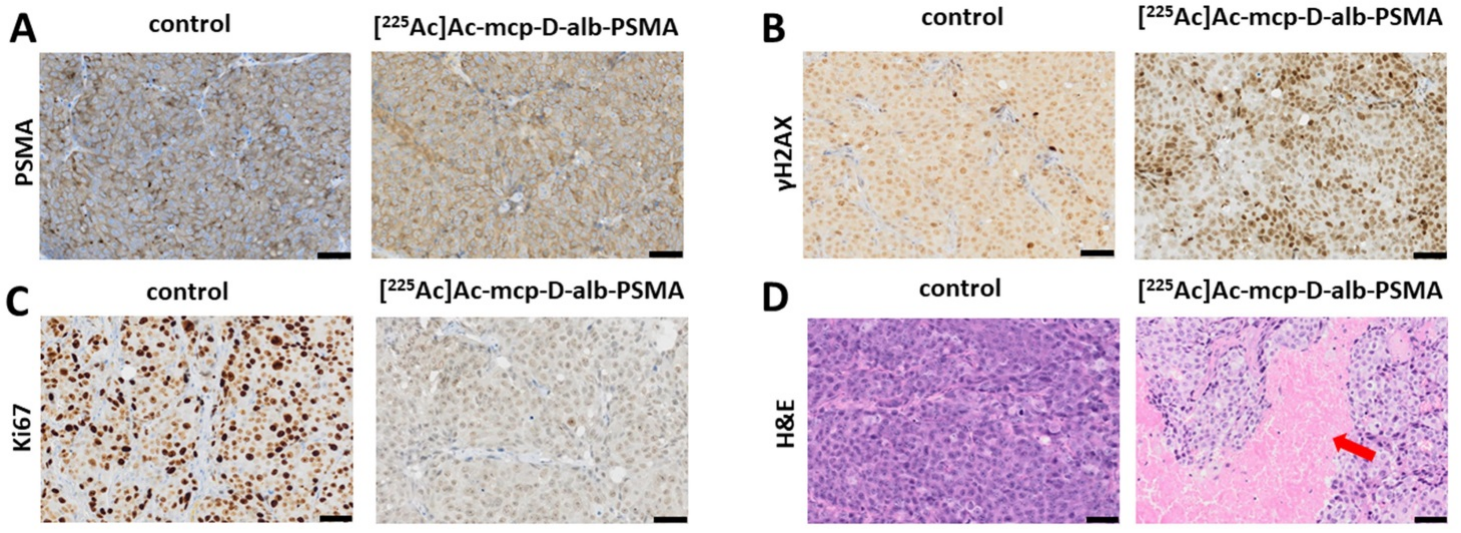
Representative histological images of tumor sections. (**A**) Untreated control tumor and **[^225^Ac]Ac-mcp-D-alb-PSMA** treated tumor (168 h p.i.) sections were stained with anti-PSMA, (**B**) anti-γH2AX showing DNA damage (represented by various intensity of the brown staining) in untreated control and in **[^225^Ac]Ac-mcp-D-alb-PSMA** treated (168 h p.i.) tumor, (**C**) anti-Ki67 staining indication proliferation status of untreated control and **[^225^Ac]Ac-mcp-D-alb-PSMA** treated (168 h p.i.) tumor and (**D**) H&E stain of untreated control and **[^225^Ac]Ac-mcp-D-alb-PSMA** treated (72 h p.i.) tumor depicting necrotic region (indicated with red arrow) in treated tumor. All images were acquired at 200× magnification. Scale bars represent 50 μm for all panels.

The IHC staining of γH2AX confirmed that there was no significant DNA damage in the liver samples. Similarly, H&E stain did not show any necrotic sites in liver tissue with the exception of samples from 120 h p.i. group of **[^225^Ac]Ac-mcp-M-alb-PSMA**, where the only significant necrosis was recorded. Representative histological images from the control mice as well as from mice applied with **[^225^Ac]Ac-mcp-D-alb-PSMA** are shown in Figure 6.

## Experimental Section

### Chemistry

All chemicals were purchased from commercial suppliers and used without further purification. Mass spectra (MALDI-MS) were recorded on a Bruker Autoflex Max MALDI/TOF-MS/MS system (Bruker, Bremen, Germany). TLC analyses for reaction control were performed on Merck Silica Gel 60 F_254_ TLC plates and visualized using 254 nm UV light. HPLC was performed on VWR Hitachi using analytical Zorbax 300SB-C18 column, 100 × 4.6 mm (Agilent Technologies, Waldbronn, Germany) and acetonitrile/water (0.1% TFA each) as mobile phase using a flow rate of 1 mL/min. Chromatographic separations were performed using automated flash column chromatography on Isolera Four (Biotage, Uppsala, Sweden) using silica gel cartridges (SNAP HC-Sfär; 5 g, 10 g, or 25 g) and reversed phase HPLC system Knauer Azura (Knauer, Berlin, Germany) with Zorbax 300SB-C18 semi-preparative column (Agilent Technologies, Waldbronn, Germany) and acetonitrile/water (0.1% TFA each) as mobile phase using a flow rate of 6 mL/min. Compound **1** as well as **mcp-M-click** and **mcp-D-click** were synthesized in accordance to the literature 25.

#### Synthesis of compound** 2**

Fmoc-L-Lys(N_3_)-OH (143 mg, 0.36 mmol), EDC∙HCl (57 mg, 0.30 mmol), HOBt∙H_2_O (67 mg, 0.41 mmol) DIPEA (62 µL, 0.36 mmol) were dissolved in 10 mL ice-cold DMF and stirred for 30 min. Afterwards, compound **1** (200 mg, 0.24 mmol) dissolved in DMF (3 mL) was added dropwise. The reaction mixture was stirred at rt over the weekend. After TLC-control, the solvent was removed, the residue dissolved in dichloromethane and washed three times (2 x H_2_O, 1 x brine). The organic phase was removed and the remaining compound was dissolved in acetonitrile/piperidine (10 mL, 1/1) and stirred for 1 h at rt to cleave the Fmoc-group. Afterwards, the solvents were removed under reduced pressure and the residue was dissolved in chloroform and washed two times with water. The organic phase was dried over Na_2_SO_4_ and the solvent was removed. The crude product was purified by automated column chromatography (chloroform/ethanol; 100/0 → 75/25). Compound **2** was obtained as yellow oil (90 mg; 0.10 mmol; 38%). Analytical HPLC: t_R_ = 14.2 min. MALDI-MS: *m/z* = 1000 [M+Na]^+^, 1016 [M+K]^+^.

#### Synthesis of compound **3**

Fmoc-Glu(tBu)-OH (90 mg, 0.21 mmol), EDC∙HCl (33 mg, 0.17 mmol), HOBt∙H_2_O (46 mg, 0.30 mmol) DIPEA (70 µL, 0.40 mmol) were dissolved in 10 mL of ice-cold DMF and stirred for 30 min. Afterwards, compound **2** (90 mg, 0.10 mmol) dissolved in DMF (3 mL) was added dropwise. The reaction mixture was stirred at room temperature overnight. After TLC-control, the solvent was removed the residue was dissolved in chloroform and washed two times with water and dried over Na_2_SO_4_ afterwards. The organic phase was removed and the remaining substance was dissolved in acetonitrile/piperidine (10 mL, 1/1) and stirred for 1 h at rt to cleave the Fmoc-group. Afterwards, the solvents were removed, the residue was dissolved in chloroform and washed two times with water. The organic phase was dried over Na_2_SO_4_ and the solvent was removed. The crude product was purified by automated column chromatography (chloroform/ethanol; 100/0 → 70/30). Compound **3** was obtained as yellow oil (100 mg; 0.09 mmol; 89%). Analytical HPLC: t_R_ = 14.9 min. MALDI-MS: *m/z* = 1186 [M+Na]^+^, 1202 [M+K]^+^.

#### Synthesis of compound **4**

4-(*p*-Iodophenyl)butyric acid (50 mg, 0.3 mmol), EDC∙HCl (27 mg, 0.14 mmol), HOBt∙H_2_O (29 mg, 0.19 mmol) and compound **3** (100 mg, 0.10 mmol) were dissolved in ice-cold DMF (10 mL). The reaction mixture was stirred at rt overnight. After TLC-control, the solvents were removed, the residue was dissolved in chloroform and washed two times with water. The organic phase was dried over Na_2_SO_4_ and the solvent was removed. The crude product was purified by automated column chromatography (chloroform/ethanol; 100/0 → 90/10). Compound **4** was obtained as yellow oil (95 mg; 0.07 mmol; 76%). Analytical HPLC: t_R_ = 16.6 min. MALDI-MS: *m/z* = 1458 [M+Na]^+^, 1474 [M+K]^+^.

#### Synthesis of compound **5**

Compound **4** (95 mg, 0.070 mmol) was dissolved in chloroform (2 mL), TFA (2 mL) was added and the mixture was stirred at rt overnight. The solvents were removed under reduced pressure and the product was precipitated by adding 20 mL of ice-cold diethyl ether. The precipitate was washed with ice-cold pentane, ice-cold chloroform, again with ice-cold diethyl ether and dried. Compound **5** was obtained as pale-yellow solid (73 mg, 0.066 mmol, 94%). Analytical HPLC: t_R_ = 12.8 min. MALDI-MS: *m/z* = 1083 [M-I]^+^, 1233 [M+Na]^+^, 1249 [M+K]^+^. HRMS (ESI, qToF): *m/z* calcd for C_83_H_109_IN_14_O_23_: 1796.6835 [M]^+^; found: 1796.6902.

#### Synthesis mcp-M-alb-PSMA

Compound **5** (22 mg, 18 µmol), **mcp-M-click** (13 mg, 23 µmol), [(CH_3_CN)_4_Cu]PF_6_ (0.6 mg, 2.3 µmol) and THPTA (1.0 mg, 2.3 µmol) were dissolved in DMF (2 mL) and stirred at rt overnight. The progress of the reaction was monitored by analytical HPLC. After completion of the reaction the solvents were removed. Excess of copper was removed by CuS-precipitation (addition of 10 mg Na_2_S). The crude product was purified by semi-preparative HPLC (20-80% acetonitrile in H_2_O + 0.1% TFA) and **mcp-M-alb-PSMA** was obtained as colorless solid (6.8 mg, 3.8 µmol, 17%). Analytical HPLC: t_R_ = 10.3 min. MALDI-MS: *m/z* = 1798 [M+H]^+^. HRMS (ESI, qToF): *m/z* calcd for C_140_H_182_I_2_N_24_O_38_: 3061.1136 [M]^+^; found: 3061.1418.

#### Synthesis mcp-D-alb-PSMA

Compound **5** (33 mg, 28 µmol), **mcp-D-click** (9 mg, 14 µmol), [(CH_3_CN)_4_Cu]PF_6_ (0.7 mg, 2.8 µmol) and THPTA (1.1 mg, 2.8 µmol) were dissolved in DMF (2 mL) and stirred at rt overnight. The progress of the reaction was followed by analytical HPLC and the solvents were removed. Excess copper was removed by CuS precipitation (addition of 10 mg Na_2_S). The crude product was purified by semi-preparative HPLC (20-80% acetonitrile in H_2_O + 0.1% TFA) and **mcp-D-alb-PSMA** was obtained as colorless solid (14 mg, 4.6 µmol, 33%). Analytical HPLC: t_R_ = 11.6 min. MALDI-MS: *m/z* = 3065 [M+H]^+^.

### HRMS Measurements

The peptide samples were recovered in 200 µL of 50% acetonitrile with 1% FA. One µL of the sample was mixed with 40 µL of 50% acetonitrile with 1% FA comprising 500 fmol/µL peptide HK1 (m/z 625.3612, z = 2+) as an internal standard. Samples were ionized by Nano-ESI with a Nanomate Triversa Ion Source at 1.7 kV and mass spectra were acquired on an impact HD mass spectrometer operated in positive mode. At least 20 spectra were averaged. Exported spectra were calibrated with the internal standard peptide by linear, single point calibration in the mMass V5.5 software 45.

### Radiolabeling and Conjugate Stability

^225^Ac was commercially supplied by ITM as [^225^Ac]AcCl_3_. Radiolabeling was performed using 100 kBq ^225^Ac, the respective ligand stem solution (10^-2^ M - 10^-6^ M) to reach the required ligand concentration (10^-3^ M - 10^-7^ M), the necessary amount of 2,5-dihydroxybenzoic acid (final concentration 0.1 M) and a volume of 0.2 M NH_4_OAc (pH 6) to adjust a total volume of 100 µL. Labeling for biological evaluation were modified and a molar activity (regarding the total ligand amount) of 5 MBq/nmol was adjusted. Every reaction mixture remained for 1 h (to keep the method constant, reaction finished after 15 min) at room temperature in a thermomixer at 350 rpm.

Once the labeling reaction was finished, samples were taken out and the ^225^Ac-radioconjugates were analyzed via TLC (two systems: 1 - 0.05 M EDTA (pH 7) on silica plates; 2 - 70/30 acetonitrile/H_2_O on alugram RP-CN plates) and analytical HPLC on a Jasco HPLC system with GABI analyzer (Elysia Raytest) for gamma detection on a Phenomenex Phenyl-Hexyl column in water/acetonitrile (0.1% TFA each). TLC plates were imaged at least 4 h later to ensure that initial decay products do not interfere and only ^225^Ac-related signals are displayed.

1-Octanol/water distribution coefficients (log D) were determined by applying the shake-flask-method. 20 µL of the reaction mixture were poured into 580 µL of 5xPBS (pH 7.4). Afterwards, 600 µL of 1-octanol was added and the two-phase-system was vigorously stirred for 5 min. Then, the samples were centrifuged, the phases were separated and the count rates were measured using ISOMED2160 sodium iodide crystal detector (at least 4 h after phase separation due to initial decay products).

### MST Measurements

The albumin-binding affinity was determined using a fluorescence-based competition assay as recently published 35. Data are given as mean values of three separate experiments with estimated confidence intervals (68.3%) using the error surface projection method in brackets obtained from PALMIST software 46.

### Cell Culture

The human prostate adenocarcinoma cell line LNCaP was obtained from ATCC (Manassas, VA, USA) and routinely maintained in RPMI-1640 medium (Thermo Fisher Scientific, Waltham, MA, USA) supplemented with 10% fetal calf serum (FCS, Merck KGaA, Darmstadt, Germany) as previously reported 25.

### Cell Binding Studies

Saturation binding studies to determine the equilibrium dissociation constants (K_D_) and the maximum binding capacities (B_max_) were performed as described elsewhere 25. In short, 30000 LNCaP cells were seeded in 48-well microplates (Greiner Bio-One GmbH, Frickenhausen, Germany) and cultivated for 48 h to allow cell adhesion and growth. During the experiment, cells were kept on ice and all reagents were added ice-cold. The cell culture medium was replaced by PBS (200 µL/well) and the microplates were preincubated for 30 min at 4 °C. The ^225^Ac-radioconjugates were added to the wells in a volume of 200 µL at different concentrations (15 pM to 500 nM). The nonspecific binding was assessed in parallel by blocking the LNCaP cells with a 1000-fold excess of unlabeled PSMA-617. After incubation of the microplates for 90 min on ice, the cells were washed three times with ice-cold PBS. The cells were lysed in 0.1 M NaOH containing 1% (w/v) sodium dodecyl sulfate. The activity of cell lysates was measured using an automatic gamma counter (Hidex Deutschland Vertrieb GmbH, Mainz, Germany).

### Clonogenic assay

Colony formation of LNCaP cells upon exposure to increasing activity concentrations of both ^225^Ac-radioconjugates was analyzed as previously reported 25 with slight modifications. Briefly, 6000 LNCaP cells were seeded in 6-well microplates (Greiner Bio-One GmbH, Frickenhausen, Germany) and cultivated overnight to allow cell adhesion. The cell culture medium was replaced by serum-free RPMI-1640 medium (2 mL/well) and the microplates were further incubated at 37 °C. After 1 h, five different activity concentrations (0.05, 0.5, 1, 5, and 50 kBq/mL) of the ^225^Ac-radioconjugates were added in triplicate. After 1 or 4 h of incubation at 37 °C, the supernatants were replaced by fresh RPMI-1640 medium with 10% FCS (2 mL/well) and plates were incubated at 37 °C for 8 d. Finally, the cell culture medium was discarded and colonies were stained with 0.5% crystal violet in 50% methanol (1 mL/well) for 30 min, after which the plates were rinsed three times with deionized water and subsequently air-dried. The plates were scanned with an Amersham Typhoon 5 Scanner (Cytiva Europe GmbH, Freiburg, Germany) and the colonies were counted using the Image-Quant TL software (Version 8.1, Cytiva Europe GmbH, Freiburg, Germany).

### Animal Studies

The biodistribution experiments with **[^225^Ac]Ac-mcp-M-alb-PSMA** and **[^225^Ac]Ac-mcp-D-alb-PSMA** were accomplished using LNCaP tumor-bearing mice. The SCID male mice (ENVIGO, Indianapolis, IN, USA) 8 weeks old were subcutaneously xenografted into the right flank with 10∙10^6^ LNCaP cells (ATCC, Manassas, VA, USA) mixed with Matrigel Matrix (Corning Inc., Corning, NY, USA) at a 1:1 ratio. The tumor growth was periodically monitored by caliper-based measurements. When the tumor volume reached 200-300 mm^3^ (i.e., approx. 6 weeks after the xenografting of the cells), the mice were enrolled in the biodistribution study. All experimental animals were housed in a specific-pathogen-free animal facility and all experiments with animals were performed in accordance with appropriate legal norms (Czech Law No. 246/1992) and with the approval of the Ministry of Education, Youth and Sports (MSMT-35035/2019-3) and approval of the Ethical committee of Faculty of Medicine and Dentistry, Palacky University in Olomouc. The number of animals was reduced as much as possible (n = 3-4 per group and time point) for *in vivo* experiments in order to strictly follow 3Rs principle.

For the purpose of biodistribution studies, the ^225^Ac-radioconjugates were diluted with saline. The diluted ^225^Ac-radioconjugates were applied retro-orbitally (r.o.) to the experimental animals 47 at a dose of 50 kBq per mouse corresponding to 10 pmol of the PSMA-inhibitor. The radioactivity application was carried out under 2% isoflurane anesthesia (FORANE, Abbott Laboratories, Abbott Park, IL, USA) to minimize animal suffering and to prevent animal motion. The mice were sacrificed by cervical dislocation 1, 4, 24, 48, 72, and 120 h (plus 168 h for **[^225^Ac]Ac-mcp-D-alb-PSMA**) post-injection and organs of interest (blood, spleen, pancreas, stomach, intestine, kidneys, liver, heart, lungs, muscle, bone, and tumor) were collected. The organs were weighed, and their radioactivity was counted on an automatic gamma counter. The uptake of the ^225^Ac-radioconjugates was expressed as a percentage of injected dose per gram of the corresponding organ (%ID/g). The area under the curve (AUC) was determined for both tested PSMA ligands from non-decay-corrected data obtained from the *ex vivo* biodistribution data of the tumors, kidneys, and blood.

### Immunohistochemistry

Formalin-fixed and paraffin-embedded mouse tissues were cut in 5 µm sections and immunostained with appropriate antibodies according to standard techniques 48 as well as with standard H&E staining. Antigen retrieval was performed in citrate buffer (pH 6) for all primary antibodies: Ki-67 (clone MIB1, DAKO; dilution 1:200), γH2AX (clone JBW301, Millipore; dilution 1:2500) and PSMA (clone YPSMA-1, Abcam; dilution 1:1000). The presence or absence of necrosis and its percentage in the tumor samples were evaluated by experienced pathologist. As well as protein expression was assessed semiquantitatively using the histoscore method, where the percentage of positive cells (0-100%) was multiplied by staining intensity (0-3), which resulted in a final histoscore between 0 and 300. The data were analyzed using Kruskal-Wallis test followed by Dunn *post hoc* test using GraphPad Prism version 7.05 (GraphPad Software, San Diego, CA, USA).

## Conclusion

In this study, two novel PSMA-targeting radioconjugates **[^225^Ac]Ac-mcp-M-alb-PSMA** and **[^225^Ac]Ac-mcp-D-alb-PSMA** comprising 4-(*p*-iodophenyl)butyrate residues as albumin binder were synthesized and successfully radiolabeled under mild conditions. The binding to LNCaP cells as well as to albumin was proven still showing a binding to the PSMA receptor in the low nM range and additionally a binding to albumin in the low µM range. The prolonged blood circulation of these radioligands resulted in greatly enhanced tumor uptake and retention over time combined with favorable tumor-to-background ratios. Histological examinations for both radioconjugates revealed no necrosis in organs (H&E staining) except the tumor tissue, but an increased DNA damage (IHC staining of γH2AX) in the tumor. Due to its substantially lower kidney and spleen accumulation, the radioconjugate **[^225^Ac]Ac-mcp-M-alb-PSMA** possessing only one PSMA- and albumin-binding unit represents a promising candidate for the treatment of metastatic castration-resistant prostate cancer as well as for further theranostic applications in combination with ^133^La as diagnostic match.

## Supplementary Material

Supplementary figures and table.

## Figures and Tables

**Table 1:** *In vitro* data of the 4-(p-iodophenyl)butyrate-containing radioconjugates **[^225^Ac]Ac-mcp-M-alb-PSMA** and **[^225^Ac]Ac-mcp-D-alb-PSMA** in comparison to **[^225^Ac]Ac-mcp-M-PSMA** and **[^225^Ac]Ac-mcp-D-PSMA** without albumin-binding entities.

Radio-conjugate	K_D_ [HSA] (µM)	K_D_ [PSMA] (nM)	B_max_ (pmol/mg of protein)	EC_50_ (kBq/mL) for 1 h treatment
**[^225^Ac]Ac-mcp-M-PSMA**	330 (240 to 460)^*^	30.83 (16.44 to 56.72)^#^	0.15 (0.12 to 0.19)^#^	2.037 (1.323 to 3.130)^#^
**[^225^Ac]Ac-mcp-D-PSMA**	150 (100 to 210)^*^	10.20 (9.52 to 10.94)^#^	1.55 (1.52 to 1.58)^#^	0.1011 (0.0668 to 0.1530)^#^
**[^225^Ac]Ac-mcp-M-alb-PSMA**	27 (19 to 37)^*^	23.38 (18.97 to 28.72)^#^	0.46 (0.43 to 0.49)^#^	0.5233 (0.4010 to 0.6767)^#^
**[^225^Ac]Ac-mcp-D-alb-PSMA**	15 (10 to 22)^*^	11.56 (10.20 to 13.08)^#^	1.96 (1.89 to 2.04)^#^	0.0519 (0.0353 to 0.0753)^#^

One experiment which was performed in triplicate. ^#^95% confidence interval.^ *^68.3% confidence interval.

**Table 2:** Area under the curve (AUC) calculated from non-decay corrected biodistribution data of **[^225^Ac]Ac-mcp-M-alb-PSMA** and **[^225^Ac]Ac-mcp-D-alb-PSMA** and the ratios of AUCs.

Radio-conjugate	AUC [%ID/g.h]			
	Tumor	Blood	Kidneys	Liver
**[^225^Ac]Ac-mcp-M-alb-PSMA**	3494 ± 194	132 ± 7.2	2269 ± 288	119 ± 4.6
**[^225^Ac]Ac-mcp-D-alb-PSMA**	6455 ± 411	249 ± 10.4	4519 ± 193	328 ± 15.8
	**AUC_Tu_-to-AUC_Bl_**	**AUC_Tu_-to-AUC_Ki_**	**AUC_Tu_-to-AUC_Li_**	
**[^225^Ac]Ac-mcp-M-alb-PSMA**	26	1.5	29	
**[^225^Ac]Ac-mcp-D-alb-PSMA**	26	1.4	20	

The data of AUCs are presented as mean values ± SD with n=4 for [^225^Ac]Ac-mcp-M-alb-PSMA and n=3 for [^225^Ac]Ac-mcp-D-alb-PSMA.
