# Supplementary material for: Modulating the pharmacokinetic profile of Actinium-225-labeled macropa-derived radioconjugates by dual targeting of PSMA and albumin
Source: Theranostics. 2022 Oct 17;12(17):7203–15. doi: 10.7150/thno.78043 (PMC9691366; doi:10.7150/thno.78043)
Supplement: Supplementary file 1 — Supplementary figures and table. [file thnov12p7203s1.pdf]

Supporting Information

Modulating the pharmacokinetic profile of Actinium-225-labeled macropa-derived radioconjugates by dual targeting of PSMA and albumin

Falco Reissig[1,2], Kristof Zarschler[1,*], Zbynek Novy[3,*], Milos Petrik[3], Katerina Bendova[3], Daniela Kurfurstova[4], Jan Bouchal[4], Marie-Charlotte Ludik[1], Florian Brandt[1,2], Klaus Kopka[1,2], Marta Khoylou[3], Hans-Jürgen Pietzsch[1], Marian Hajduch[3] and Constantin Mamat[1,2,*]

[1] Helmholtz-Zentrum Dresden-Rossendorf, Institute of Radiopharmaceutical Cancer Research, Bautzner Landstraße 400, D-01328 Dresden, Germany.

[2] Technische Universität Dresden, Faculty of Chemistry and Food Chemistry, D-01062 Dresden, Germany.

[3] Palacky University Olomouc, Faculty of Medicine and Dentistry, Institute of Molecular and Translational Medicine and Czech Advanced Technology and Research Institute, Hnevotinska 1333/5, 779 00 Olomouc, Czech Republic.

[4] Palacky University Olomouc, Faculty of Medicine and Dentistry, Institute of Clinical and Molecular Pathology, Hnevotinska 976/3, 775 15 Olomouc, Czech Republic.

Table of Contents

MALDI-TOF MS spectra of compounds S1

HRMS spectra of final compounds S5

Radio-TLC and HPLC-chromatograms for stability testing S7

Colony forming assay - clonogenic activity of LNCaP cells S8

Fluorescence-based competition assay to determin albumin binding S9

Histological assessment of organ sections S11

Biodistribution data for both radioconjugates S12

MALDI-TOF MS spectra of compounds

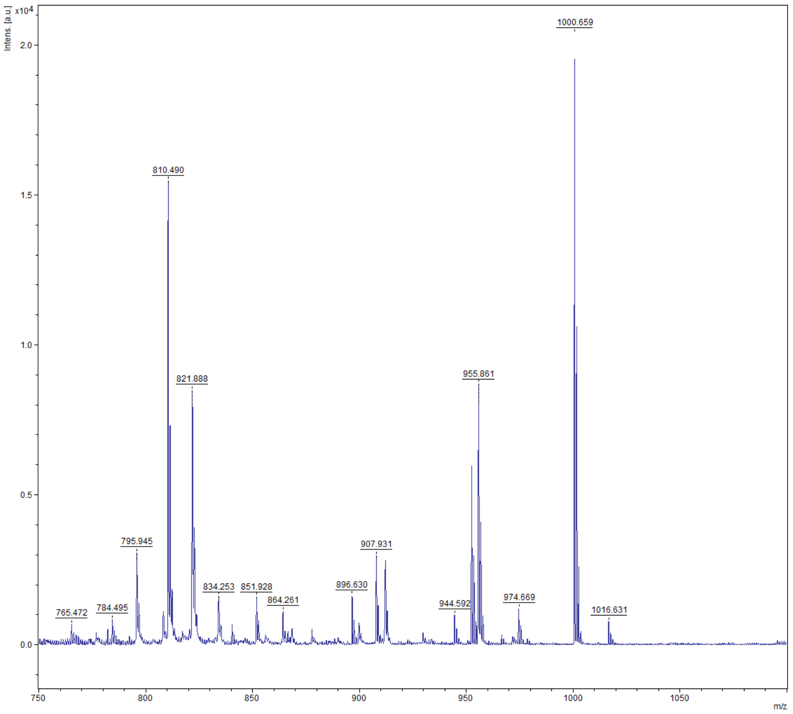

Figure S1. MALDI-TOF MS spectrum of compound **2**.

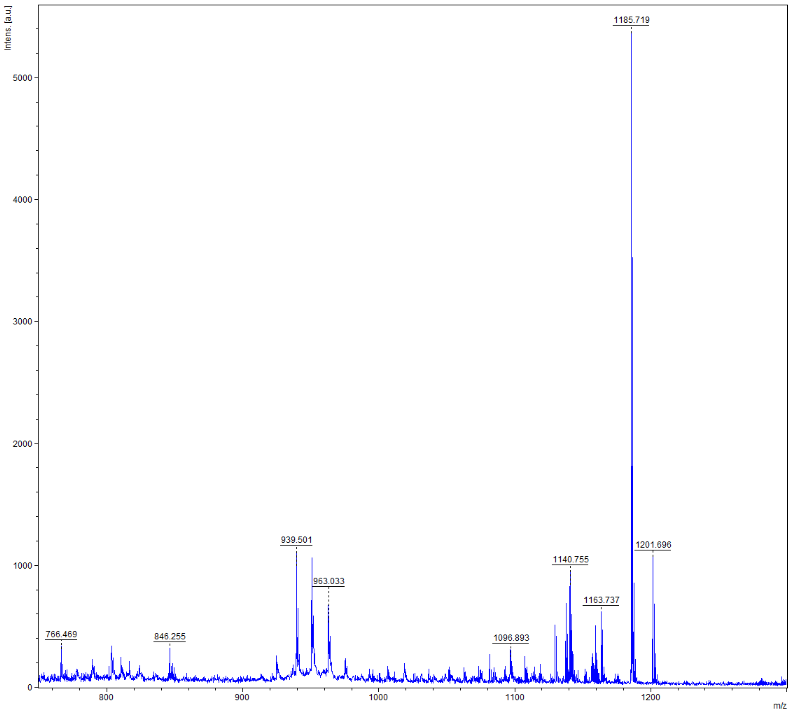

Figure S2. MALDI-TOF MS spectrum of compound **3**.

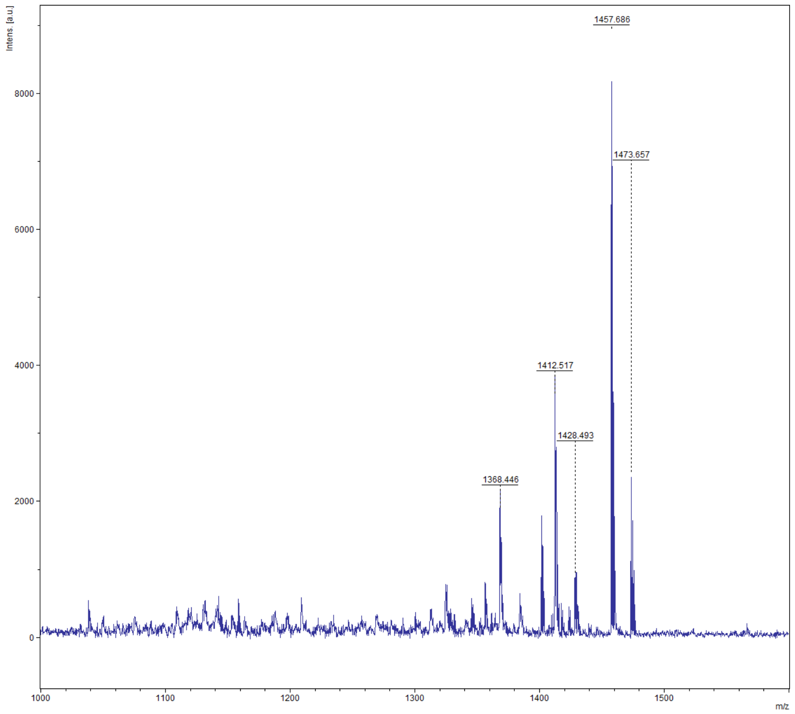

Figure S3. MALDI-TOF MS spectrum of compound **4**.

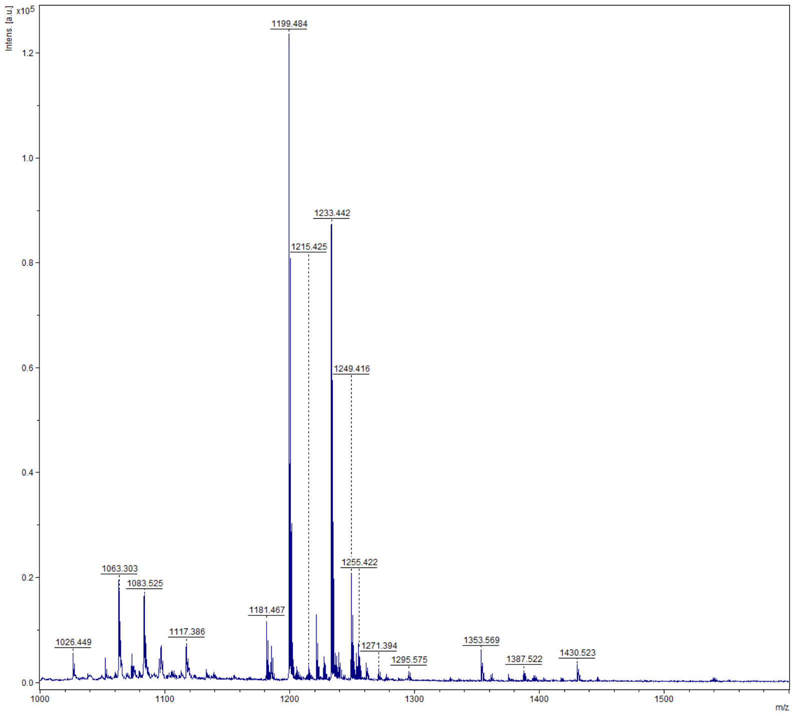

Figure S4. MALDI-TOF MS spectrum of compound **5**.

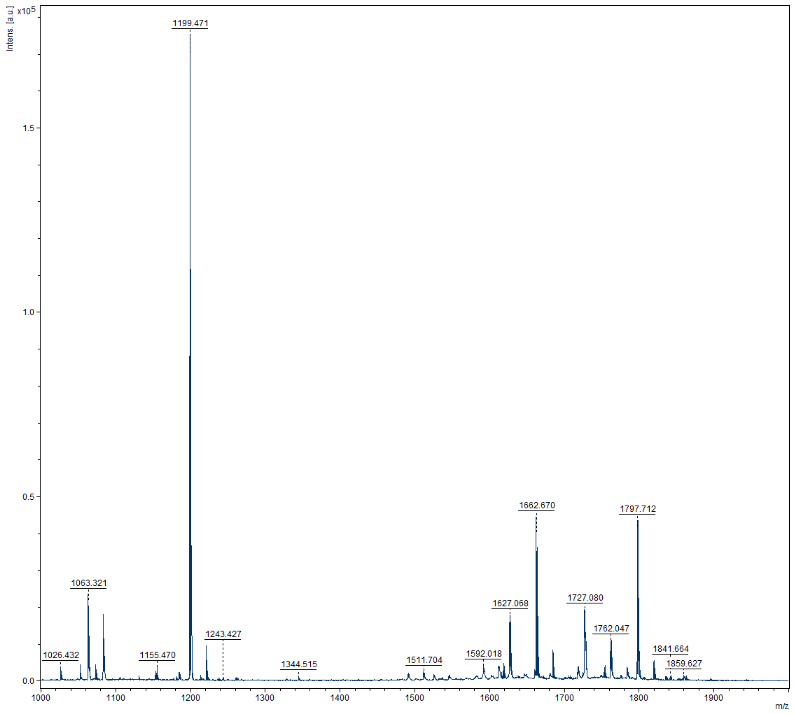

Figure S5. MALDI-TOF MS spectrum of compound **mcp-M-alb-PSMA**.

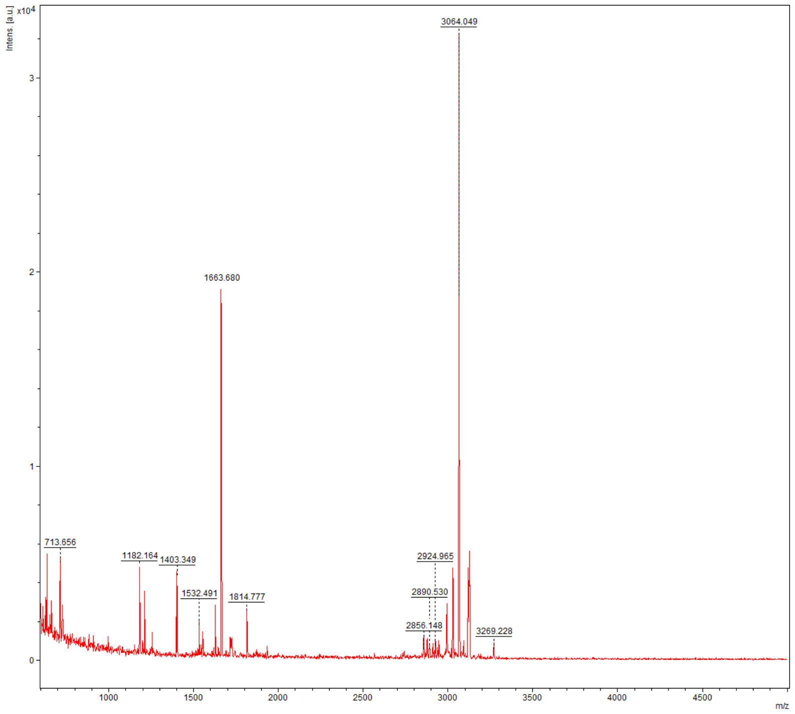

Figure S6. MALDI-TOF MS spectrum of compound **mcp-D-alp-PSMA**.

HRMS Spectra of compounds

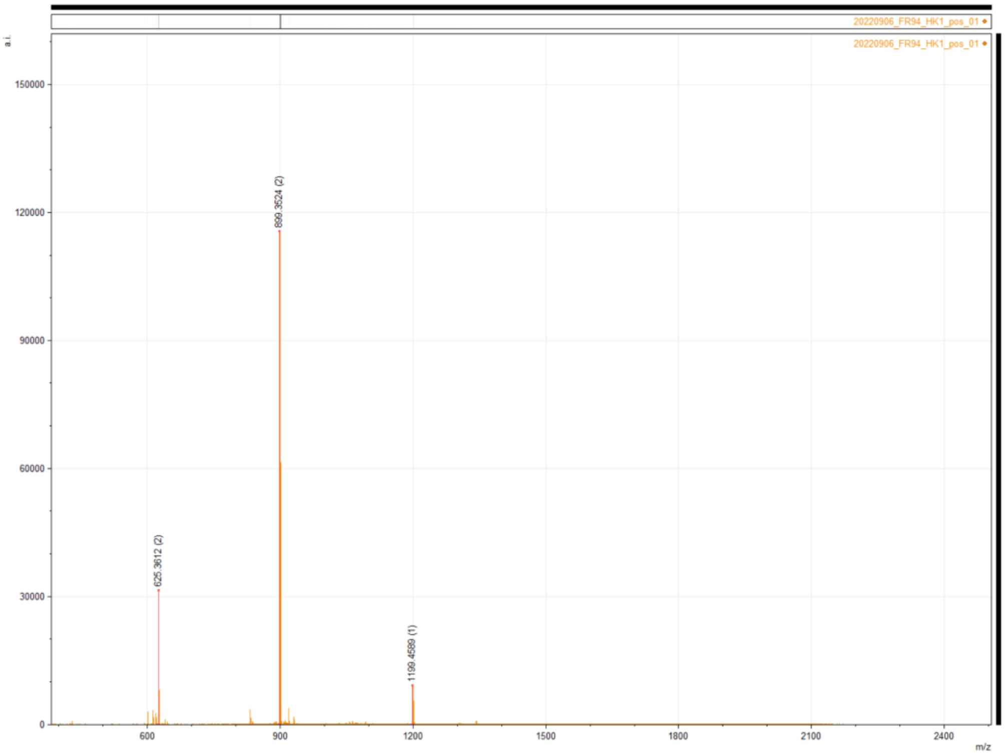

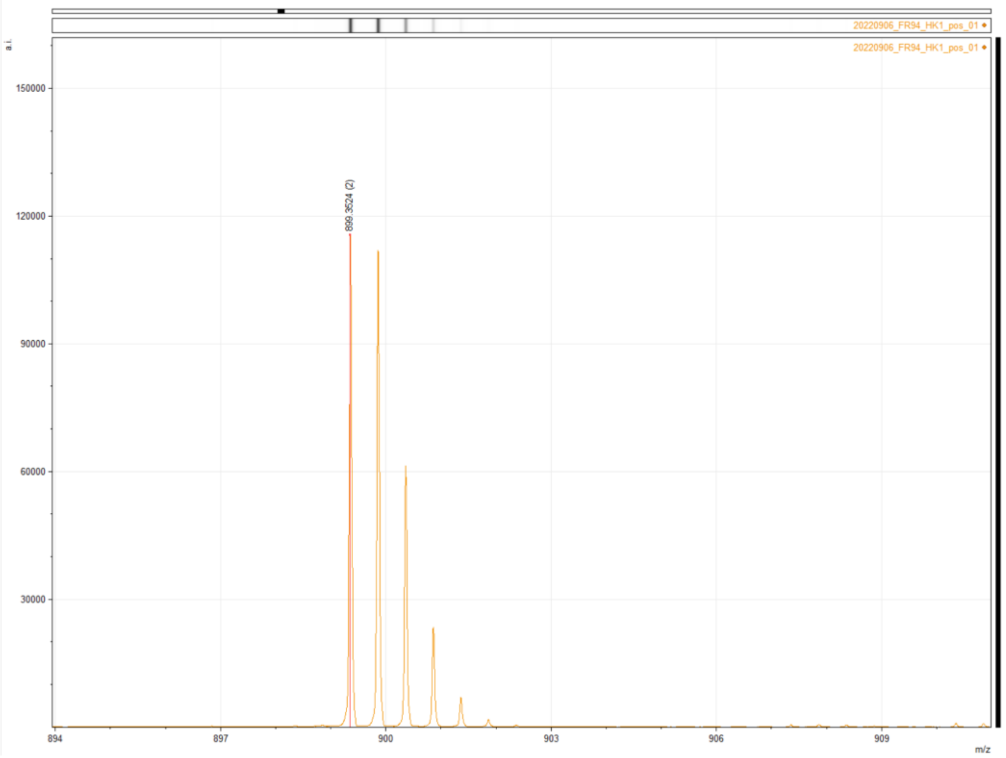

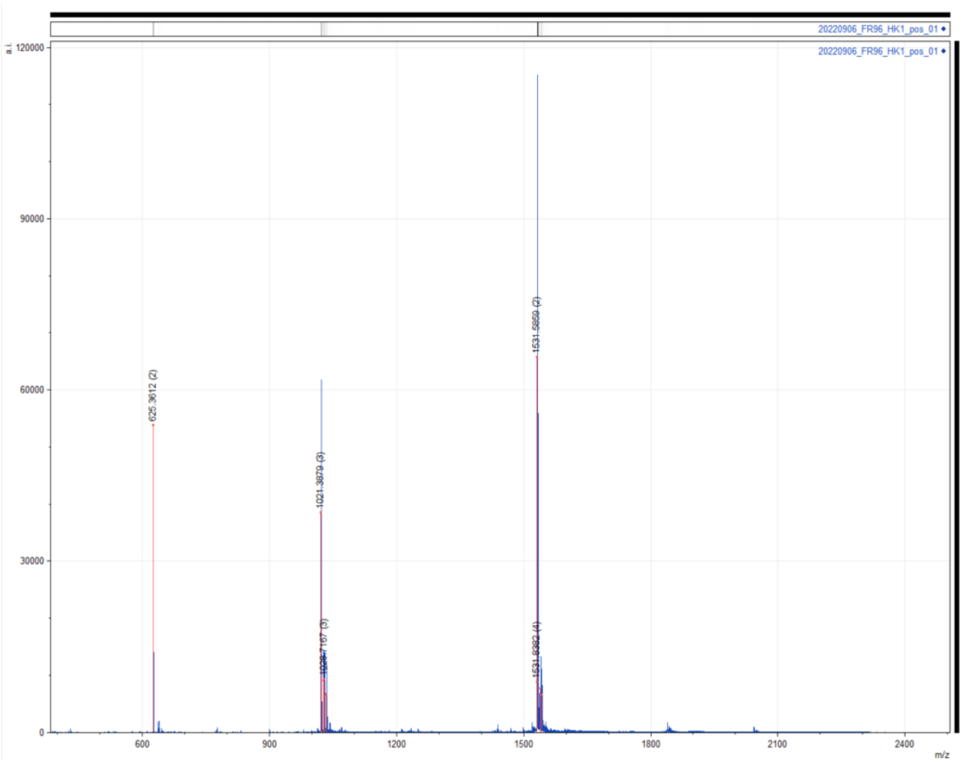
20220906_FR96_HK1_pos_01
a.i.
625.3612 (2)
1021.3879 (3)
1028.7167 (3)
1531.5859 (2)
1531.8382 (4)
m/z

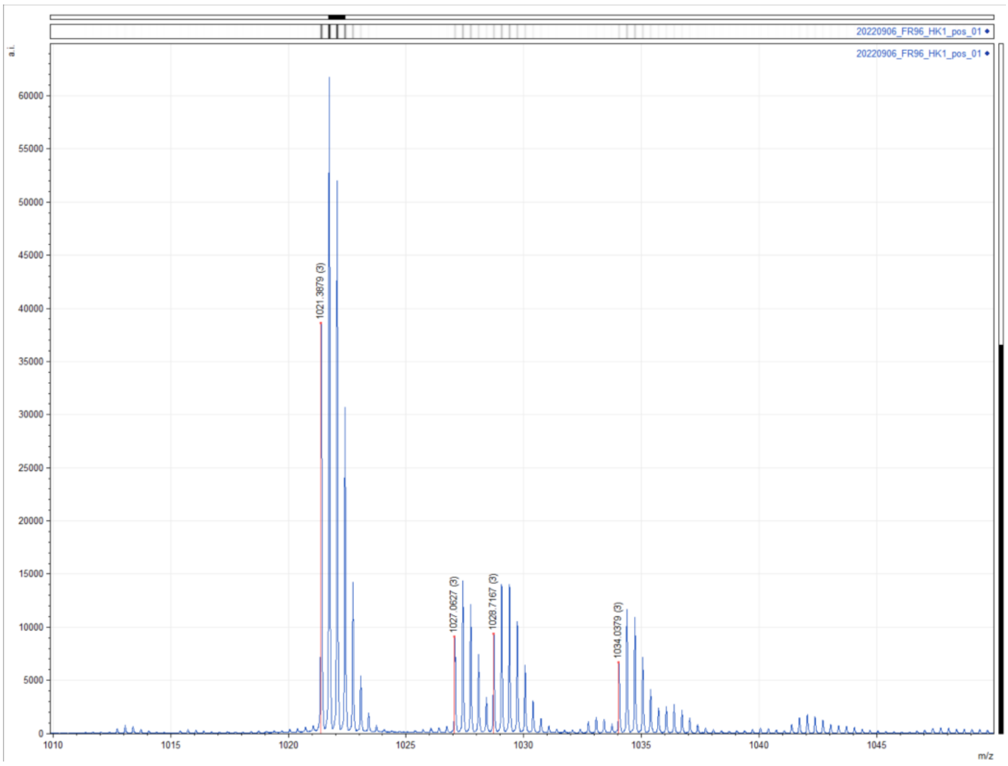
20220906_FR96_HK1_pos_01
a.i.
1021.3879 (3)
1027.0627 (3)
1028.7167 (3)
1034.0379 (3)
m/z

Radio-TLC and HPLC-chromatograms for stability testing

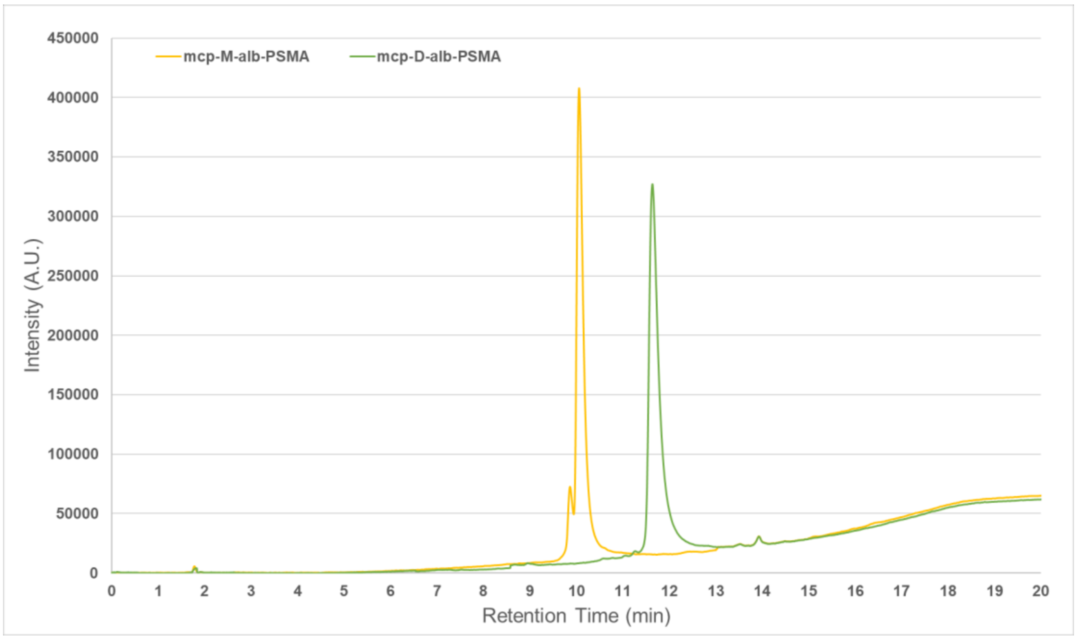

Figure S7. HPLC-chromatograms of **mcp-M-alb-PSMA** and **mcp-D-alb-PSMA**

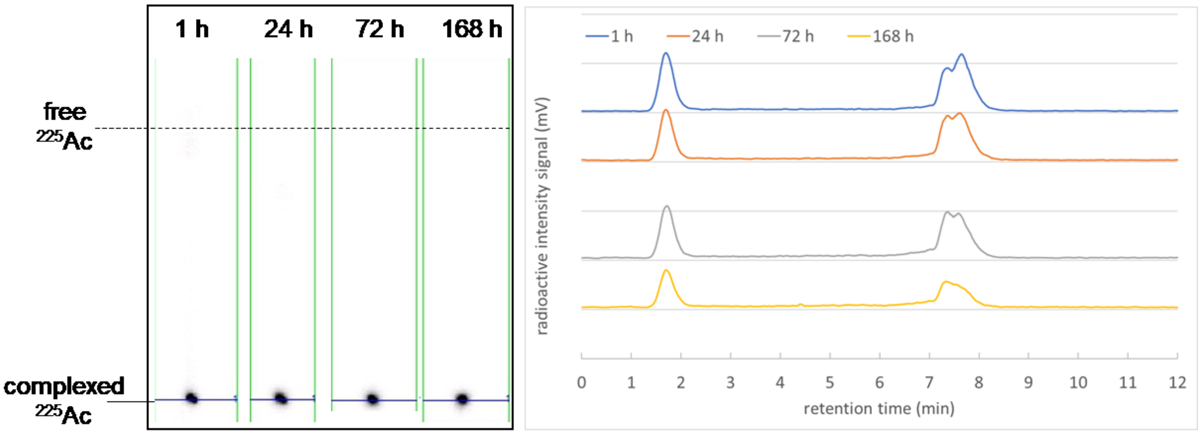

Figure S8. TLC- and HPLC-chromatograms for stability assay of **mcp-M-alb-PSMA**

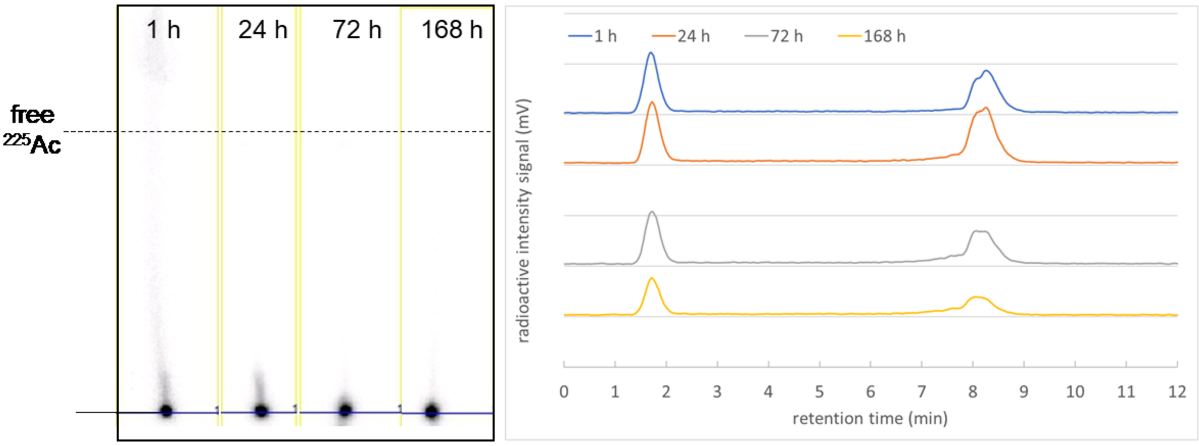

Figure S9. TLC- and HPLC-chromatograms for stability assay of **mcp-D-alb-PSMA**

Colony forming assay - clonogenic activity of LNCaP cells

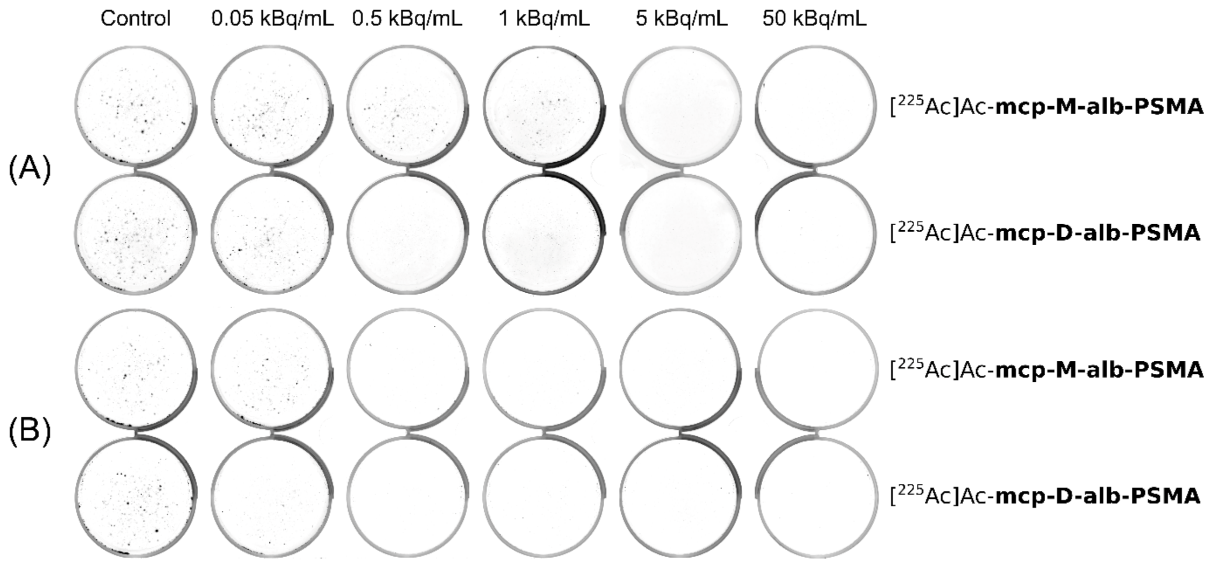

Figure S10. Colony formation of LNCaP cells in response to treatment with ^{225}Ac-labeled PSMA ligands for (A) 1 h or (B) 4 h, respectively. Log phase cells were plated and 24 h later treated with different activity concentrations of **[^{225}Ac]Ac-mcp-M-alb-PSMA** or **[^{225}Ac]Ac-mcp-D-alb-PSMA**. After the indicated treatment time, cells were supplemented with fresh medium and cultures were incubated for eight days to allow colony formation.

Fluorescence-based competition assay to determin albumin binding

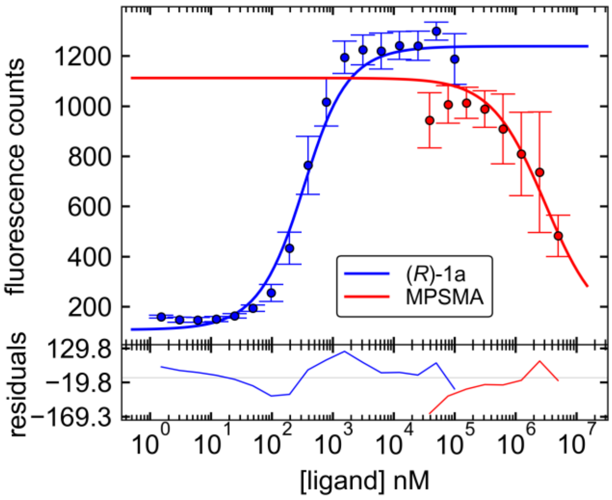

Figure S11. Albumin-binding curve for **mcp-M-PSMA**. Data points for **(*R*)-1a** (blue) are the averaged fluorescence counts (*n* = 6) for the direct titration to HSA. Data points for **mcp-M-PSMA** (red) are the averaged fluorescence counts (*n* = 3) for the competition experiment.

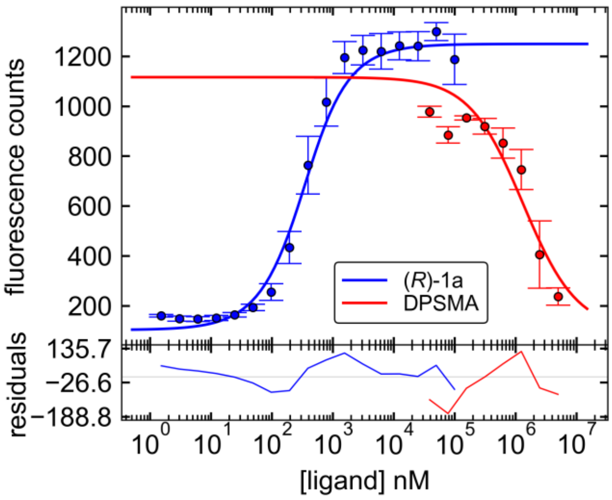

Figure S12. Albumin-binding curve for **mcp-D-PSMA**. Data points for **(*R*)-1a** (blue) are the averaged fluorescence counts (*n* = 6) for the direct titration to HSA. Data points for **mcp-D-PSMA** (red) are the averaged fluorescence counts (*n* = 3) for the competition experiment.

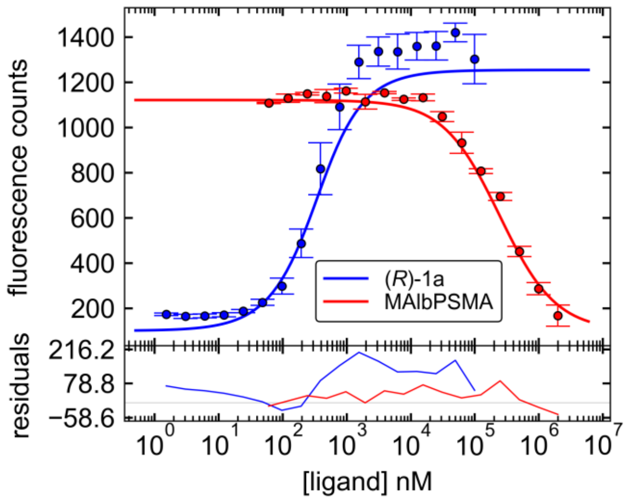

Figure S13. Albumin-binding curve for **mcp-M-alb-PSMA**. Data points for **(*R*)-1a** (blue) are the averaged fluorescence counts (*n* = 6) for the direct titration to HSA. Data points for **mcp-M-alb-PSMA** (red) are the averaged fluorescence counts (*n* = 3) for the competition experiment.

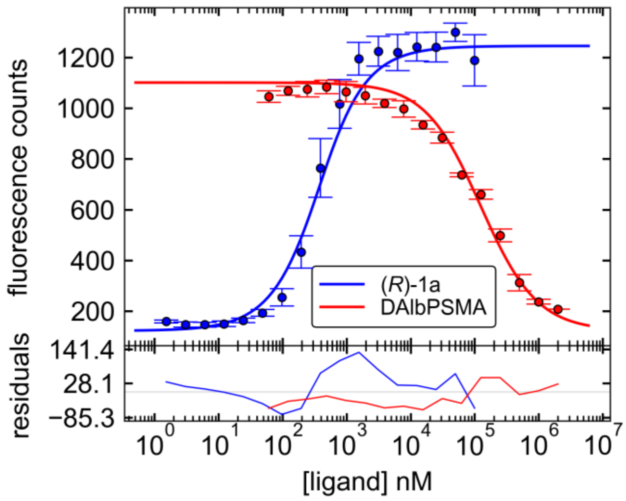

Figure S14. Albumin-binding curve for **mcp-D-alb-PSMA**. Data points for **(*R*)-1a** (blue) are the averaged fluorescence counts (*n* = 6) for the direct titration to HSA. Data points for **mcp-D-alb-PSMA** (red) are the averaged fluorescence counts (*n* = 3) for the competition experiment.

Histological assessment of organ sections

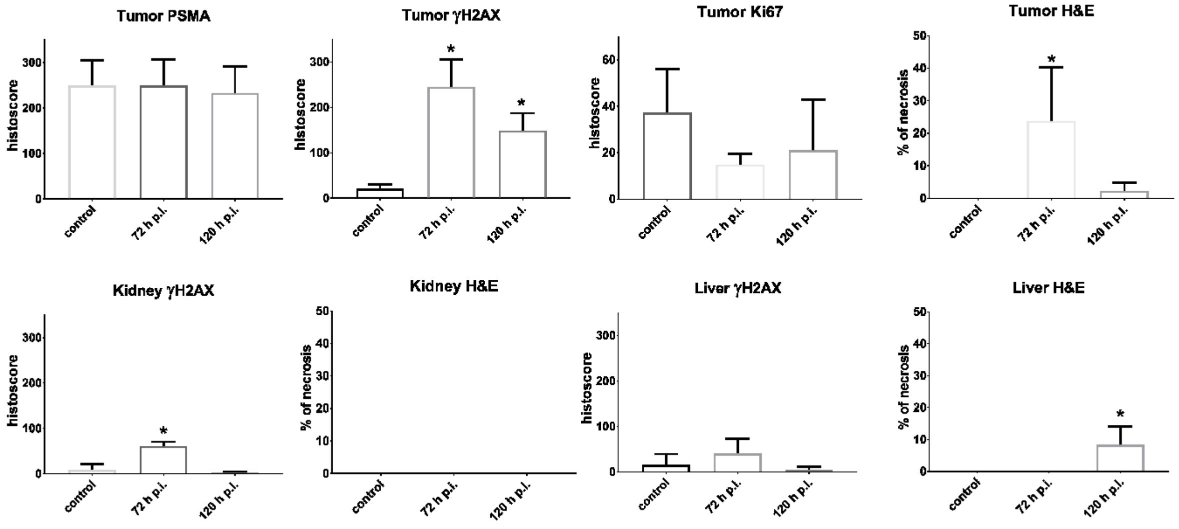

Figure S15. Histological assessment of organ sections. The graphs describe quantification of the corresponding immunohistochemical (IHC) staining of the tumor/kidney/liver tissues from the control group (untreated) and from groups injected with **[^{225}Ac]Ac-mcp-M-alb-PSMA** sacrificed in 72 and 120 h post injection (p.i.) respectively. Values are expressed as means with standard deviations. P-values $p < 0.05$ are indicated with *. n = 6 for control groups and n= 3-4 for **[^{225}Ac]Ac-mcp-M-alb-PSMA** treated groups.

Biodistribution data for both radioconjugates

Table S1. Biodistribution data of ^{225}Ac-labeled, 4-(*p*-iodophenyl)butyrate-containing PSMA radioconjugates in LNCaP tumor-bearing mice from 1 h to 120 resp. 168 h after injection. The values are indicated as average (AV) ± standard deviation (SD) obtained from each group of mice (n = 3−4) and listed as percentage of injected dose per gram of tissue [%ID/g].

	**1 h p.i.**		**4 h p.i.**		**24 h p.i.**		**48 h p.i.**		**72 h p.i.**		**120 h p.i.**		**168 h p.i.**	
	**[^{225}Ac]Ac-mcp-M-alb-PSMA**													
	**AV**	**SD**	**AV**	**SD**	**AV**	**SD**	**AV**	**SD**	**AV**	**SD**	**AV**	**SD**	**AV**	**SD**
blood	6.47	0.93	4.80	0.56	0.54	0.29	0.08	0.02	0.03	0.01	0.03	0.02	N.D.	N.D.
spleen	7.24	2.61	3.40	0.92	1.24	0.15	1.31	0.47	1.12	0.09	1.43	0.16	N.D.	N.D.
pancreas	0.57	0.10	0.57	0.06	0.26	0.04	0.10	0.01	0.06	0.03	0.07	0.03	N.D.	N.D.
stomach	0.83	0.05	0.58	0.09	0.39	0.19	0.17	0.03	0.10	0.04	0.11	0.03	N.D.	N.D.
intestine	0.60	0.07	0.54	0.09	0.81	0.41	0.10	0.01	0.12	0.04	0.06	0.01	N.D.	N.D.
kidneys	29.50	2.91	67.94	20.67	33.08	11.59	15.74	3.73	9.59	2.49	8.82	4.40	N.D.	N.D.
liver	1.44	0.15	1.43	0.15	1.08	0.17	1.19	0.08	1.08	0.13	1.27	0.12	N.D.	N.D.
heart	2.03	0.31	1.63	0.15	0.34	0.03	0.22	0.06	0.15	0.01	0.23	0.10	N.D.	N.D.
lung	6.27	1.42	4.99	2.00	2.19	0.48	1.77	1.37	1.47	1.17	1.73	0.88	N.D.	N.D.
muscle	0.53	0.05	0.51	0.06	0.14	0.01	0.07	0.04	0.08	0.03	0.12	0.13	N.D.	N.D.
femur	0.61	0.10	0.62	0.19	0.32	0.15	0.44	0.15	0.32	0.13	0.43	0.39	N.D.	N.D.
tumor	5.96	1.64	17.98	4.23	29.47	5.74	36.49	1.73	38.72	6.74	46.04	4.77	N.D.	N.D.
	**[^{225}Ac]Ac-mcp-D-alb-PSMA**													
	**AV**	**SD**	**AV**	**SD**	**AV**	**SD**	**AV**	**SD**	**AV**	**SD**	**AV**	**SD**	**AV**	**SD**
blood	8.44	0.94	6.50	0.74	2.85	0.33	1.22	0.24	0.70	0.18	0.20	0.04	0.11	0.04
spleen	7.11	2.20	6.21	1.67	5.24	1.60	8.33	1.35	10.20	0.25	10.00	1.20	11.42	2.89
pancreas	0.68	0.11	0.58	0.05	0.50	0.06	0.35	0.07	0.39	0.04	0.42	0.10	0.37	0.03
stomach	0.98	0.15	0.59	0.15	0.61	0.13	0.40	0.14	0.27	0.04	0.34	0.12	0.27	0.06
intestine	0.80	0.16	0.64	0.06	0.58	0.07	0.37	0.09	0.26	0.05	0.19	0.02	0.17	0.02
kidneys	9.23	1.39	21.79	2.37	43.72	6.84	59.90	6.46	50.10	5.75	37.64	2.54	33.67	9.57
liver	2.42	0.47	2.45	0.35	2.52	0.46	3.73	0.63	3.39	0.46	3.90	0.16	4.31	0.67
heart	2.47	0.50	2.32	0.20	1.21	0.12	0.94	0.28	0.82	0.08	0.88	0.05	0.83	0.27
lung	5.67	0.44	4.57	1.17	2.30	0.28	2.75	1.10	2.98	1.13	7.50	5.29	4.95	4.37

muscle	0.49	0.07	0.58	0.19	0.28	0.05	0.29	0.11	0.20	0.05	0.24	0.04	0.22	0.08
femur	0.84	0.19	0.89	0.37	0.74	0.13	0.96	0.29	0.87	0.16	1.22	0.25	1.30	0.49
tumor	3.35	0.85	10.56	2.32	27.58	4.31	57.39	4.14	86.39	17.15	120.04	8.58	126.92	4.75
